# Intracellular Accumulation of IFN-λ4 Induces ER Stress and Results in Anti-Cirrhotic but Pro-HCV Effects

**DOI:** 10.3389/fimmu.2021.692263

**Published:** 2021-08-23

**Authors:** Olusegun O. Onabajo, Fang Wang, Mei-Hsuan Lee, Oscar Florez-Vargas, Adeola Obajemu, Chizu Tanikawa, Joselin M. Vargas, Shu-Fen Liao, Ci Song, Yu-Han Huang, Chen-Yang Shen, A. Rouf Banday, Thomas R. O’Brien, Zhibin Hu, Koichi Matsuda, Ludmila Prokunina-Olsson

**Affiliations:** ^1^Laboratory of Translational Genomics, Division of Cancer Epidemiology and Genetics, National Cancer Institute, Bethesda, MD, United States; ^2^Institute of Clinical Medicine, National Yang Ming Chiao Tung University, Taipei, Taiwan; ^3^Institute of Medical Science, The University of Tokyo, Tokyo, Japan; ^4^Institute of Statistical Science, Academia Sinica, Taipei, Taiwan; ^5^Department of Epidemiology, School of Public Health, Nanjing Medical University, Nanjing, China; ^6^Institute of Biomedical Sciences, Academia Sinica, Taipei, Taiwan; ^7^Infections and Immunoepidemiology Branch, Division of Cancer Epidemiology and Genetics, National Cancer Institute, Rockville, MD, United States; ^8^Graduate School of Frontier Sciences, The University of Tokyo, Tokyo, Japan

**Keywords:** *IFNL4*, HCC (hepatocellular carcinoma), liver cirrhosis, ER stress, HCV (Hepatitis C)

## Abstract

*IFNL3/IFNL4* polymorphisms are inversely associated with the risk of chronic hepatitis C virus (HCV) infection and cirrhosis, two major risk factors for developing hepatocellular carcinoma (HCC). To further explore these inverse associations and their molecular underpinnings, we analyzed *IFNL3/IFNL4* polymorphisms represented by the *IFNL4* genotype (presence of rs368234815-dG or rs12979860-T alleles) in HCV patients: 2969 from Japan and 2931 from Taiwan. *IFNL4* genotype was associated with an increased risk of HCV-related HCC (OR=1.28, 95%CI=1.07-1.52, P=0.0058) in the general population of Japanese patients, but not in Taiwanese patients who achieved treatment-induced viral clearance. *IFNL4* genotype was also associated with a decreased risk of cirrhosis (OR=0.66, 95%CI=0.46-0.93, P=0.018, in Taiwanese patients). We then engineered HepG2 cells to inducibly express IFN-λ4 in the presence or absence of interferon lambda receptor 1 (IFNLR1). Induction of IFN-λ4 resulted in its intracellular accumulation, mainly in lysosomes and late endosomes, and increased ER stress, leading to apoptosis and reduced proliferation. We identified the very-low-density lipoprotein receptor (*VLDLR*), which facilitates HCV entry into hepatocytes, as a transcript induced by IFN-λ4 but not IFN-λ3. Our results suggest that the molecular mechanisms underlying the anti-cirrhotic but pro-HCV associations observed for *IFNL3/IFNL4* polymorphisms are, at least in part, contributed by intracellular accumulation of IFN-λ4 causing ER stress in hepatic cells.

## Introduction

Infection with hepatitis C virus (HCV) persists in a chronic stage in 75-85% of infected individuals; 10-20% of these patients progress to liver cirrhosis and then to hepatocellular carcinoma (HCC), the main type of primary liver cancer, with a rate of 1-4% per year (1). Genetic polymorphisms within the *IFNL3*/*IFNL4* genomic region have been identified as the strongest predictors of spontaneous and treatment-induced clearance of HCV infection (2–5).

Due to strong linkage disequilibrium (LD) in the *IFNL3*/*IFNL4* region (6), many markers provide similar genetic associations, including rs368234815 that controls IFN-λ4 production (7), rs4803217 that was suggested to affect IFN-λ3 stability (8), and rs12979860, a non-functional polymorphism in the first intron of *IFNL4* (3). Among these, a dinucleotide polymorphism, rs368234815-TT/dG, is the most informative marker for predicting HCV clearance in all populations (6, 7). Individuals with the rs368234815-dG allele have a frameshift in the first exon of the *IFNL4* gene, which creates an open reading frame for interferon lambda 4 (IFN-λ4), a type III IFN. Homozygous carriers of the rs368234815-TT allele, including most Asians (~90%) and Europeans (~50%), but only a minority of individuals of African ancestry (~10%), are natural IFN-λ4 knockouts (6).

Carriers of the *IFNL4*-dG allele are more likely to develop chronic HCV infection and fail to respond to HCV treatment. Given that chronic HCV infection is a strong risk factor for HCC, the *IFNL4*-dG allele would be expected to be associated with the risk of HCC as well. However, associations between *IFNL3/IFNL4* polymorphisms and HCC risk have been inconsistent and reported by some studies (9–13), but not others (14, 15). Surprisingly, the alleles associated with poor HCV clearance were also associated with reduced risk of liver fibrosis (16, 17), which is an aberrant wound-healing response to chronic liver injury and a pre-stage of liver cirrhosis (18). Thus, the interplay between the *IFNL3/IFNL4* polymorphisms, HCV infection, and the risk of liver cirrhosis and HCC remains unclear. Here, we addressed these questions using genetic, genomic, and functional tools.

## Materials and Methods

### HCV+ Patients From BioBank Japan

A total of 2969 individuals were from BioBank Japan, which between 2003 and 2007 recruited ~200,000 patients with 47 common diseases from 12 Japanese medical institutes representing 67 hospitals (19). From the BioBank Japan database, we selected all the patients who were HCV-RNA positive at the time of recruitment and ascertained for primary HCC status (yes/no) based on histology, imaging, and laboratory tests. No information about HCV treatment and outcomes was available. The project was approved by the ethical committees of the University of Tokyo, and all participants provided written informed consent. Genomic DNA from peripheral blood was genotyped with an Illumina HumanOmniExpressExome BeadChip or a combination of the Illumina HumanOmniExpress and HumanExome BeadChips (20). The genotypes were prephased with MACH (21) and imputed with Minimac using the 1000 Genomes Project Phase 1 (version 3) East Asian reference panel (22). *IFNL4*-rs12979860 was imputed with high confidence, and association analysis was conducted using a logistic regression model using imputed gene dosage, adjusting for age, sex, and the top 2 principal components (PCs).

### HCV+ Patients From REVEAL II Cohort, Taiwan

The REVEAL-II (Risk Evaluation of Viral Load Elevation and Associated Liver Diseases) clinical cohort has been described (23). Briefly, treatment-naive HCV+ patients without HCC were enrolled in the prospective study during 2004–2014. The patients were treated with Peg-IFN and ribavirin (RBV) for 48 or 24 weeks (for patients with HCV genotype 1 and other genotypes, respectively), and sustained virologic response (SVR) was determined from the serum HCV RNA test results 24 weeks post-treatment. Patients who didn’t achieve an SVR were re-treated with the same regimen, and the combined SVR was estimated based on the treatment and retreatment.

Demographic and clinical data were obtained *via* chart reviews with standardized forms. Cirrhosis at baseline (before treatment) was determined by abdominal ultrasound (in 81.6% of patients) or liver biopsy (in 18.4%) and categorized as yes/no. DNA samples were genotyped for *IFNL4*-rs368234815 by a custom TaqMan genotyping assay, as previously described (7). Only individuals with available information about baseline cirrhosis and *IFNL4*-rs368234815 genotypes were included in the analysis (n=2,931). Incident HCC diagnoses after treatment completion were verified by medical records and electronic linkage with the Taiwan National Cancer Registration and the National Death Certification databases.

The person-years of follow-up for each patient were calculated from the enrolment date to either the date of HCC identification, the date of death, or December 31, 2014, whichever came first. The incidence rates of HCC per 1,000 person-years were calculated by dividing the number of newly developed HCC cases by person-years of follow-up. Multivariable Cox proportional hazards models were used to examine the outcomes in relation to *IFNL4* genotypes (as 0 and 1), controlling for several clinical predictors including age, sex, baseline liver cirrhosis, serum alanine aminotransferase (ALT), HCV genotype (genotype 1 *vs.* other), and SVR. Hazard ratios (HRs) with 95% confidence intervals (CIs) and statistical significance levels were determined by a two-sided p-value of 0.05. The proportionality assumption of Cox models was examined, and the assumption was not violated. All analyses were performed using the SAS statistical software package (version 9.1; SAS Institute Inc., Cary, NC, USA).

### HBV+ Patients From China

HBV-positive HCC patients (n=1,300) were consecutively recruited between 2006 and 2010 in Nantong and Nanjing, China (24). Age and sex-matched controls were recruited from the same geographical areas and included 1,344 HBV persistent carriers that were positive for both HBsAg and antibody against hepatitis B core antigen (anti-HBc), as well as 1,344 subjects with natural clearance of HBV-negative for HBsAg but positive for antibodies against hepatitis B surface antigen (anti-HBs) and anti-HBc. All the cases and controls were negative for HCV antibody (anti-HCV). No information about HBV treatment and outcomes was available. Genomic DNA was extracted from blood leukocytes with phenol-chloroform. DNA samples were genotyped for *IFNL4*-rs368234815 by a custom TaqMan genotyping assay, as previously described (7). Association analysis was conducted using a logistic regression model adjusting for relevant covariates as indicated.

### Human Cells Lines and Primary Cells

Human cell lines: HepG2 (hepatoma) and 293T (embryonal kidney) were acquired from the American Type Culture Collection (ATCC), while LX-2 (hepatic stellate) was purchased from Millipore-Sigma. Stable HepG2 cell lines expressing doxycycline (dox)-inducible GFP-tagged IFN-λ3 and IFN-λ4 (IFN-λ3-GFP and IFN-λ4-GFP cells, respectively), have been described (25). All cell lines were maintained in DMEM supplemented with 10% FBS (Invitrogen), penicillin, and streptomycin; 5 μg/ml blasticidin and 1 mg/ml neomycin were additionally used for the stable cell lines. Expression of IFN-λ3–GFP and IFN-λ4–GFP was induced by 0.5 μg/ml or specified concentrations of dox for indicated time points. LX-2 cells were maintained in DMEM supplemented with 2% FBS, 100 units/ml penicillin, 100 µg/ml streptomycin, and 2 nM glutamine media. Primary human hepatic stellate cells (HSCs) were purchased from ScienCell Research Laboratories and maintained in stellate cell media with 10% FBS and stellate cell growth supplement (ScienCell). All cell lines were tested bi-monthly for mycoplasma contamination using the MycoAlert Mycoplasma Detection kit (Lonza) and were authenticated annually using the AmpFLSTR Identifiler Plus Kit (ThermoFisher Scientific) by the Cancer Genomics Research Laboratory (CGR, NCI). Fresh primary human hepatocytes (PHHs) from 12 anonymous donors purchased from BioreclamationIVT and genotyped for *IFNL4*-rs368234815 were previously described (25).

### Generation of IFNLR1 Knockout Cell Line Using CRISPR/Cas9 Genome Editing

Exon 3 of the NM_170173.3 transcript, which is the first exon common for all four alternative isoforms of *IFNLR1*, was selected as a target region for designing six candidate gRNAs with a CRISPR design tool (http://crispr.mit.edu/). Double-stranded oligonucleotides were cloned into the linearized SmartNuclease vector (EF1-T7-hspCas9-T2A-RFP-H1-gRNA) with a red fluorescent protein (RFP, System Biosciences) and IFNLR1-guide RNA (IFNLR1-gRNA) plasmids were validated by DNA sequencing. 293T cells were transiently transfected in 6-well plates with six individual IFNLR1-gRNA plasmids using Lipofectamine 3000 (Life Technologies). The cells were harvested 4 days post-transfection and subjected to fluorescence-activated cell sorting (FACS) for RFP with a FACS Aria III (BD Biosciences). Genomic DNA from sorted RFP-positive cells was extracted using DNeasy Blood & Tissue Kit (Qiagen) and used for sequencing the exon 3 target region. One out of 6 IFNLR1-gRNA plasmids was selected as the most optimal (**Figure S1A**) and was transfected into IFN-λ4-GFP cells in a 12-well plate, together with linearized screening puromycin marker (Clontech). Two days post-transfection, cells were plated in a 10-cm plate, and fresh media containing puromycin (2 µg/ml) was added to the plate two days later. After three weeks of selection, visible clones were picked and transferred into 24-well plates with fresh media without puromycin. DNA was extracted from each of the clones and sequenced as described above. Final clones represented the stable IFN-λ4-GFP-IFNLR1^KO^ cells. Potential off-target sites were predicted with the online tool CCTop (http://crispr.cos.uni-heidelberg.de) (26). The top ten predicted sites were tested by sequencing the genomic DNA of IFN-λ4-GFP-IFNLR1^KO^ cells, and no off-target mutations were detected (data not shown).

### Interferon-Stimulated Response Element Luciferase Reporter (ISRE-Luc) Assays

Cells were seeded in 96-well plates (2 × 10^4^ cells/well) and reverse-transfected with 100 ng/well of Cignal ISRE-Luc reporter (Qiagen) using Lipofectamine 3000. After 24 hours of transfection, cells were treated for 8 hours in triplicates with recombinant IFNα (0.5 ng/ml, PBL), custom IFN-λ3 (20 ng/ml) and IFN-λ4 (50 ng/ml) (25), or were induced for 12 or 24 hours with dox (0.5 µg/ml), with untreated cells used as a negative control. Induction of ISRE-Luc reporter was evaluated by measuring Firefly and Renilla luciferase activity with dual-luciferase reporter assays on Glomax-Multi Detection System (Promega). The results were presented as relative light units (RLU), corresponding to Firefly normalized by Renilla luciferase activity.

### Interferon Treatment

Cells were seeded in 12-well plates and treated for 8 hours in biological quadruplicates with IFNα (0.5 ng/ml, R&D Systems), IFNβ (0.5 ng/ml, GenScript), IFNγ (1 ng/ml, R&D Systems), IFN-λ1 (5 ng/ml, R&D Systems), IFN-λ2 (60 ng/ml, R&D Systems), and custom IFN-λ3 (20 ng/ml) and IFN-λ4 (50 ng/ml) (25), with no treatment used as a negative control.

### Western Blotting

Cells were lysed in RIPA buffer (Sigma) supplemented with protease inhibitor cocktail (Promega) and PhosSTOP (Roche). Cell lysates were resolved on Blot 4-12% Bis-Tris gel (ThermoFisher) and transferred to nitrocellulose membranes with iBlot 2 Dry Blotting System (ThermoFisher). The membranes were probed with primary antibodies against IFNLR1 (#NBP1-84381, Novus Biologicals), STAT1 (#9172, Cell Signaling Technology), phospho-STAT1 (Tyr701, #58D6, Cell Signaling Technology), IFN-λ4 (ab196984; Abcam), GAPDH (ab37168, Abcam) and HRP-linked secondary antibody, goat anti-rabbit IgG (#7074; Cell Signaling Technology) or goat anti-mouse IgG (San Cruz, sc-2031). Signal detection was done with HyGLO™ quick spray chemiluminescent HRP antibody detection reagent (Denville Scientific) and Chemidoc Touch Imaging System (BioRad) and quantified by ImageJ software.

### Confocal Microscopy

HepG2 cells were transfected for 24 hours with corresponding Halo-tagged constructs in 4-well chambered slides for fixed cells or in 4-well coverslip slides for live cells (2 × 10^5^ cells/well, LabTek). For fixed cells, a BacMam system (ThermoFisher) was used to deliver baculoviruses expressing GFP-tagged proteins targeted to specific organelles (N-acetylgalactosaminyltransferase for Golgi, Rab5a for early endosomes, Rab7a for late endosomes, and LAMP1 for lysosomes), 6 hours prior to transfection. Cells were incubated with cell-permeant TMR red Halo-tag ligand (1:2,000 for 15 min, Promega) 24 hours post-transfection, fixed with 4% paraformaldehyde, mounted with Prolong Gold antifade mounting media with DAPI (ThermoFisher) and coverslip.

For live cells, media in chambered slides was replaced with live-cell imaging solution (Life Technologies), supplemented with 20 mM glucose, 24 hours post-transfection. Cell-permeant TMR red Halo-tag ligand was added to cells (1:2,000 for 15 min). Imaging was done on an LSM700 confocal laser scanning microscope (Carl Zeiss) using an inverted oil lens at 40x magnification; live imaging was done using a temperature and CO2-controlled chamber, with 6 sec scans every minute for 12 hours.

### Tunicamycin Treatment and Sendai Virus (SeV) Infection

HepG2 cells were treated with or without tunicamycin (20 µg/ml, Sigma) for 24 hours. For SeV infection, PHHs and HSCs were infected with SeV (7.5 × 10^5^ chicken embryo ID50 [CEID50] per milliliter, Charles River Laboratories) and collected at the indicated time points. All experiments were done in triplicates.

### RNA Extraction and Quantitative Reverse Transcriptase-Polymerase Chain Reaction (qRT-PCR) Analysis

Total RNA was extracted using an RNeasy Mini Kit with on-column DNase digestion (Qiagen). RNA quantity and quality were evaluated by NanoDrop 8000 (Thermo Scientific) and Bioanalyzer 2100 (Agilent Technologies). RNA integrity numbers (RIN) of all RNA samples were > 9.5. cDNA was synthesized from the total RNA with the RT^2^ First Strand Kit (Qiagen), with an additional DNase I treatment step. qRT-PCR assays were performed in technical quadruplicates in 384-well plates on QuantStudio 7 instrument (Life Technologies), with RT² SYBR Green (Qiagen) or TaqMan (Thermo Fisher) expression assays). The expression of target genes was normalized by geometric means of endogenous controls (*GAPDH* and *ACTB*), presented as ΔCt values. The 2^-ΔΔCT^ method, in which ΔΔCt = ΔCt (experiment) – ΔCt (control), and fold = 2^-ΔΔCt^ was used to calculate the relative abundance of target mRNA expression and fold change. Data are shown as mean ± SEM based on biological replicates.

### Cell Proliferation Assays

For proliferation assays, HepG2 cells were induced with 0.5µg/ml dox and treated with 10 μM 5-bromo-2′-deoxyuridine (BrdU) for 3 h. Dead cells were detected with a near-IR Live/Dead kit (ThermoFisher) and stained using a PE BrdU flow kit (BD Biosciences). For coculture experiments and proliferation experiments, HepG2 cells were labeled with Far Red dye (ThermoFisher). Cells were analyzed with multiparametric flow cytometry on a FACS Aria III (BD Biosciences) and FlowJo.v10 software (BD Biosciences).

### Cell Viability, Apoptosis, and Colony Formation Assays

Cells seeded in 96-well plates (8000 cells/well) were mock- or dox-induced (0.5 µg/ml) in quadruplicates for 24, 48, and 72 hours, then subjected to cell viability assays using CellTiter-Glo assays (Promega) and cell apoptosis assays using ApoTox-Glo Triplex assay (Promega). Luminescence was measured on the GloMax-multi detection system (Promega). For the colony formation assays, cells (2,000 cells/well) were mock- or dox-induced (0.5 µg/ml) in triplicates in 6-well plates for two weeks. The colonies were stained with 0.5% crystal violet, photographed, and counted using ImageJ software. Cell cycle was evaluated using PI staining and analyzed on FlowJo.10 software (BD Biosciences). Seeded cells were fixed with 70% ethanol on ice for 2 hours, washed twice with PBS, and treated with RNAse A (100µg/ml, Qiagen). Cells were then stained with PI (20µg/ml) for 10 minutes and were immediately analyzed on an Accuri C6 (BD Biosciences).

### RNA Sequencing (RNA-Seq)

Total RNA was extracted from cells using an RNeasy Mini Kit with an on-column DNase digestion (Qiagen) and quantitated using Qubit 4 Fluorometer (ThermoFisher). Ribosomal RNA was depleted with Ribo-Zero treatment, then 60 ng was used for library preparation using KAPA Stranded RNA-Seq Library Preparation Kit (Illumina, KR0934, v-1.13). The samples were barcoded and pooled in sets of eight per run to generate paired 150-bp reads with a NextSeq 550 Sequencing System (Illumina), generating 21.2 - 118.8 million reads per sample. Quality control analysis of RNA-seq data was carried out with the FastQC pipeline. Paired RNA-seq reads were aligned to the ENSEMBL human reference genome GRCh37.75 (hg19) with STAR v 2.5.3a (27) using default parameters, and BAM files were generated with SAMtools v 1.5 (28). Data normalization and analysis of differential gene expression were done with DESeq2 R package v 1.22.2 (29, 30). Thresholds of fold change ≥ 1.5 (log2 fold change = 0.58) and false-discovery rate (FDR) adjusted P-value < 0.05 were used to identify differentially expressed genes (DEG) between experimental conditions. Identified DEGs were analyzed with Ingenuity Pathway Analysis (IPA) software (Qiagen). The generated RNA-seq dataset for 108 samples (54 individual samples in duplicates) has been deposited to NCBI Gene Expression Omnibus (GEO) with an accession number GSE145038.

### Statistical and Bioinformatic Analyses

Analysis of experimental data: Experimental data were analyzed using a two-sided, unpaired Student’s t-test, and P-values <0.05 were considered statistically significant. Statistical significance of the RNA-seq differential expression data was determined using Wald negative binomial test with Benjamini–Hochberg adjustment for multiple testing (30). Unless otherwise specified, data plotting and statistical analyses were performed with Prism 7 (GraphPad), SPSS v.25 (IBM), or R packages. Means are presented with standard errors (SEM) based on biological replicates.

## Results

### In HCV Patients *IFNL4* Genotype Is Associated With Protection From Liver Cirrhosis but Does Not Affect HCC Risk in Patients With Viral Clearance

First, we evaluated progression to HCC in 2969 HCV-infected patients from the BioBank Japan (19), using an intronic *IFNL4* marker rs12979860, which is completely linked with rs368234815 in Asians (r^2^ = 1.0). The *IFNL4*-rs12979860-T allele (a proxy for *IFNL4-dG*, which supports the production of IFN-λ4) was associated with an increased risk of HCC (per-allele OR=1.28, p=0.0058, **Table 1**). Because HCV-positive patients were selected from the general population, this association likely represents patients who have never been treated as well as those who failed to clear HCV after treatment, but these questions were not explored due to a lack of information on treatments and outcomes.

**Table 1:** *IFNL4* genotype is associated with increased risk of HCC in HCV-infected patients in Japan.

Cases: HCV+ patients with HCC		Controls: HCV+ patients without HCC or cirrhosis		OR (95% CI)#	p-value#
N	rs12979860 T-allele, %	N	rs12979860 T-allele, %		
1002	15.5	1967	13.8	1.28 (1.07-1.52)	0.0058

^#^per-risk allele, adjusting for age, sex, and two main principal components. Reference allele: rs12979860-C corresponds to IFNL4-rs368234815-TT, TT/TT – IFN-λ4 is not produced.

We then analyzed the clinical REVEAL II cohort from Taiwan (23), which included 2,931 HCV-infected patients monitored for progression to HCC after treatment with peg-IFNα/RBV (**Table 2**). We genotyped *IFNL4*-rs368234815, which has a direct functional effect on IFN-λ4 production (7, 25). Since the rs368234815-dG allele is uncommon in Asian populations (<7% in 1000 Genomes Project), we used a dominant genetic model, combining all carriers of the dG allele (TT/dG and dG/dG) in one group, designated as the *IFNL4* genotype group. In the REVEAL II cohort, carriers of *IFNL4* genotype, as expected (2, 4), had reduced sustained virologic response (SVR) after treatment or retreatment with IFNα/RBV (OR=0.30, p=1.63E-18, **Table 3**). Among all REVEAL II patients, *IFNL4* genotype was associated with progression to HCC (OR=1.79, p=0.011, **Table 4**), but this association was not significant after accounting for SVR after treatment or retreatment (**Tables 4**, **5**). A context-dependent variant, rs117648444 G->A, alters amino acid 70 within exon 2 from Serine to Proline only in the presence of rs368234815-dG allele (*IFNL4* transcript), and results in functionally weak IFNλ4-S70 instead of strong IFNλ4-P70 protein (6, 7, 31). The rs117648444-A allele is relatively common in European (12%) and African (7.5%) ancestries, where it has a statistically significant modifying effect on associations of rs368234815-dG allele (31–33). However, this allele has <0.5% frequency in East-Asian populations (based on five reference populations from the 1000 Genomes Project), precluding further analyses based on the combination of rs368234815 (rs12979860) and rs117648444 in patients from Japan and Taiwan.

**Table 2:** Baseline characteristics of HCV-infected patients in the REVEAL II cohort, Taiwan.

Characteristics	Total N = 2931	Liver cirrhosis at baseline		P-value^#^
		Yes N = 508 (SD or %)	No N = 2423 (SD or %)	
Age (years)	53.46 (9.81)	56.03 (8.68)	52.92 (9.95)	<0.0001
Follow-up (years)	6.19 (4.33)	6.55 (4.06)	6.12 (4.39)	0.030
ALT, U/L	127.7 (101.1)	132.9 (94.54)	126.6 (102.3)	0.188
Platelet count, 10^3^/µL	150.1 (78.10)	116.8 (109.6)	156.8 (153.5)	<0.0001
Gender				0.0004
Male	1455 (49.64)	216 (42.52)	1239 (51.13)	
Female	1476 (50.36)	292 (57.48)	1184 (48.87)	
*IFNL4*-rs368234815				0.044
TT/TT	2618 (89.32)	468 (92.13)	2150 (88.73)	
TT/dG	303 (10.34)	40 (7.87)	263 (10.85)	
dG/dG	10 (0.34)	0 (0.00)	10 (0.41)	
SVR at initial treatment				<0.0001
No	568 (19.38)	144 (29.21)	424 (18.07)	
Yes	2271 (77.48)	349 (70.79)	1922 (81.93)	
Unknown	92 (3.14)			
SVR at any treatment*				<0.0001
Never	454 (15.49)	112 (22.72)	342 (14.58)	
Ever	2385 (81.37)	381 (77.28)	2004 (85.42)	
Unknown	92 (3.14)			
HCV genotype^$^				0.871
Genotype 1	1625 (55.44)	280 (55.12)	1345 (55.51)	
Other	1306 (44.56)	228 (44.88)	1078 (44.49)	

^#^T-test for continuous variables and Chi-squared test for categorical variables; *HCV genotype 1 vs. other genotypes (2, 3, 4, and 6); ^$^ SVR – sustained virologic response either at initial treatment or retreatment.

**Table 3:** Association of the *IFNL4* genotype with reduced SVR in HCV-infected patients in the REVEAL II cohort, Taiwan.

Genotype of *IFNL4*- rs368234815	SVR, N = 2839 N (%)		OR (95% CI), P-value Covariates in multivariable models					
	Yes	No	crude	age, sex	age, sex, ALT	age, sex, baseline cirrhosis, ALT	HCV genotype 1 age, sex, baseline cirrhosis, ALT	other HCV genotypes* age, sex, baseline cirrhosis, ALT
**Initial Treatment with peg-IFNα/RBV**								
TT/TT	2091 (82.16)	454 (17.84)	Ref	Ref	Ref	Ref	Ref	Ref
TT/dG and dG/dG	180	114	0.343	0.343	0.354	0.338	0.310	0.462
	(61.22)	(38.78)						
			(0.265-0.443)	(0.265-0.443)	(0.272-0.459)	(0.260-0.440)	(0.225-0.427)	(0.264-0.808)
			2.24E-16	2.47E-16	5.62E-15	7.33E-16	7.43E-13	0.007
**Initial Treatment and Retreatment with peg-IFNα/RBV**								
TT/TT	2193 (86.17)	352 (13.83)	Ref	Ref	Ref	Ref	Ref	Ref
TT/dG and dG/dG	192	102	0.302	0.302	0.318	0.308	0.265	0.560
	(65.31)	(34.69)						
			(0.232-0.394)	(0.231-0.395)	(0.242-0.418)	(0.234-0.405)	(0.191-0.368)	(0.296-1.060)
			9.11E-19	1.63E-18	1.68E-16	3.60E-17	1.66E-15	0.075

*Other HCV genotypes include genotypes 2, 3, 4, and 6; SVR, sustained virologic response; ALT, alanine aminotransferase.

**Table 4:** Progression to HCC in HCV-infected patients in the REVEAL II cohort, Taiwan in relation to *IFNL4* genotype and cirrhosis.

HR (95% CI), P-value Covariates in multivariable models								
Genotype of *IFNL4*- rs368234815	HCC, N (%)	Total N, person-year of follow-up	Incidence(1/1000)	Crude	age, sex	age, sex, SVR at any treatment^#^	age, sex, ALT SVR at any treatment	age, sex, ALT, HCV genotype^#^, SVR at any treatment
**All HCV patients with information on baseline cirrhosis status, n = 2931 (100%)**								
TT/TT	111 (82.84)	2618 (16279.35)	6.82	Ref	Ref	Ref	Ref	Ref
TT/dG Or dG/dG	23 (17.16)	313 (1816.60)	12.66	1.91 (1.22-2.99) P=0.0049	1.79 (1.14-2.82) P=0.011	1.38 (0.85-2.25) P=0.195	1.45 (0.89-2.36) P=0.138	1.45 (0.89-2.36) P=0.141
**HCV patients with baseline cirrhosis, N = 508 (17.3%)**								
TT/TT	51	468 (3127.33)	16.31	Ref	Ref	Ref	Ref	Ref
TT/dG Or dG/dG	10	40 (197.71)	50.58	3.30 (1.66-6.56) P=0.0007	2.32 (1.15-4.68) P=0.019	1.83 (0.88-3.79) P=0.103	2.00 (0.96-4.16) P=0.066	2.00 (0.96-4.16) P=0.066
**HCV patients without baseline cirrhosis, N = 2423 (82.7%)**								
TT/TT	60	2150 (13152.02)	4.56	Ref	Ref	Ref	Ref	Ref
TT/dG or dG/dG	13	273 (1621.89)	8.02	1.80 (0.99-3.27) P=0.055	1.80 (0.99-3.29) P=0.055	1.22 (0.62-2.41) P=0.57	1.25 (0.63-2.48) P=0.52	1.25 (0.63-2.47) P=0.53

^#^SVR – sustained virologic response either at initial treatment or retreatment with peg-IFNα/RBV; *HCV genotype 1 vs. other genotypes (2, 3, 4, and 6).

**Table 5:** Progression to HCC in HCV-infected patients in the REVEAL II cohort, Taiwan in relation to *IFNL4* genotype and SVR after treatment/retreatment with peg-IFNα/RBV.

	HR (95% CI), P-value Covariates in multivariable models							
Genotype of *IFNL4*- rs368234815	HCC, N (%)	Total N, person-year of follow-up	Incidence (1/1000)	Crude	age, sex	age, sex, baseline cirrhosis	age, sex, baseline cirrhosis, ALT	age, sex, baseline cirrhosis, ALT, HCV genotype#
HCV patients with SVR information, n=2839*								
TT/TT	109 (84.50)	2545 (15542.24)	7.01	Ref	Ref	Ref	Ref	Ref
TT/dG and dG/dG	20 (15.50)	294 (1725.00)	11.59	1.71 (1.06-2.75)	1.58 (0.98-2.56)	1.69 (1.05-2.73)	1.74 (1.07-2.83)	1.71 (1.05-2.77)
				P=0.028	P=0.060	P=0.033	P=0.024	P=0.030
**Ever SVR - at initial treatment or retreatment, N = 2385**								
TT/TT	78	2193 (13329.75)	5.85	Ref	Ref	Ref	Ref	Ref
TT/dG or dG/dG	7	192 (1171.57)	5.97	1.05 (0.49-2.28)	0.98 (0.45-2.13)	1.10 (0.51-2.40)	1.14 (0.52-2.49)	1.14 (0.52-2.49)
				P=0.90	P=0.96	P=0.81	P=0.74	P=0.74
**Never SVR - at initial treatment or retreatment, N = 454**								
TT/TT	31	352 (2212.49)	14.01	Ref	Ref	Ref	Ref	Ref
TT/dG or dG/dG	13	102 (553.42)	23.49	1.77 (0.92-3.40)	1.66 (0.86-3.18)	1.82 (0.94-3.53)	1.87 (0.96-3.64)	1.81 (0.93-3.53)
				P=0.088	P=0.13	P=0.075	P=0.065	P=0.081

*SVR status was unknown for 92 HCV patients; SVR, sustained virologic response; ALT, alanine aminotransferase; ^#^HCV genotype 1 vs. other genotypes (2, 3, 4, and 6).

*IFNL4* genotype and other markers in the *IFNL3*/*IFNL4* region have been linked with reduced hepatic inflammation and fibrosis, a pre-stage for cirrhosis (16, 17, 34), potentially mitigating progression to HCC. In the REVEAL II cohort, we also observed a lower prevalence of cirrhosis at baseline in the presence of *IFNL4* genotype (OR=0.66, p=0.018, **Table 6**). Analyses stratified according to baseline cirrhosis status showed an association of *IFNL4* genotype with HCC progression in patients with cirrhosis (OR=2.32, p=0.019, **Table 4**) but not without cirrhosis (OR=1.80, p=0.055, **Table 4**), although this association was eliminated after adjusting for SVR. *IFNL4* genotype was not associated with progression to HCC in Chinese patients with HBV (**Table 7**), in line with the absence of association between *IFNL4* genotype and HBV risk (35, 36). Thus, our results indicate that *IFNL4* genotype is moderately associated with protection from cirrhosis but not with progression to HCC after accounting for viral clearance.

**Table 6:** Prevalence of cirrhosis in HCV-infected patients in relation to *IFNL4* genotype in the REVEAL II cohort, Taiwan.

Genotype of *IFNL4*-rs368234815	Liver cirrhosis at baseline, N = 2931 N (%)		OR (95% CI), P-value	
	Yes	No	Crude	adjusted for age, sex
	N = 508	N = 2403		
	N (%)	N (%)		
TT/TT	468 (17.88)	2150 (82.12)	Ref	Ref
TT/dG or dG/dG	40 (12.78)	273 (87.22)	0.67(0.48-0.95) P=0.025	0.66 (0.46-0.93) P=0.018

TT/TT, IFN-λ4 is not produced.

**Table 7:** *IFNL4* genotype is not associated with an increased risk of HCC in HBV-infected patients in China.

Genotypes of *IFNL4*-rs368234815	HBV, HCC patients n = 1291	HBV persistent n = 1332	HBV spontaneous clearance n = 1320	OR (95% CI)^a^	*P* ^a^	OR (95% CI)^b^	*P* ^b^
	N (%)	N (%)	N (%)				
TT/TT	1128 (87.4)	1158 (86.9)	1163 (88.1)	Ref		Ref	
dG/TT	156 (12.1)	167 (12.5)	152 (11.5)	0.95 (0.75-1.21)	0.694	1.10 (0.87-1.39)	0.424
dG/dG	7 (0.5)	7 (0.5)	5 (0.4)	0.91 (0.31-2.63)	0.857	1.42 (0.45-4.50)	0.548
Dominant TT/TT *vs.* dG/TT&dG/dG	12.6	12.7	11.9	0.95 (0.76-1.20)	0.676	1.11 (0.88-1.40)	0.372
Additive, per allele							
TT	93.4	93.2	93.9	Ref		Ref	
dG	6.6	6.8	6.1	0.95 (0.77-1.19)	0.669	1.11 (0.89-1.38)	0.338

Logistic regression analyses adjusted for age, gender, smoking, and drinking status.

a HCC patients vs. HBV persistent carriers.

b HBV persistent carriers vs. HBV natural clearance subjects.

### IFN-λ4 Expression Decreases Proliferation of Hepatic Cells

Based on previous reports linking *IFNL3/IFNL4* genotypes and fibrosis risk (16, 17, 34) and our current results on association with cirrhosis risk in HCV patients, we explored molecular mechanisms that could explain these associations. Genetic variants might affect the expression and function of both IFN-λ3 and IFN-λ4, making it hard to delineate the individual contribution of these IFNs by genetic analysis alone. Thus, we explored functional effects of both IFN-λ3 and IFN-λ4 in a panel of hepatoma HepG2 cell lines, in which we inducibly expressed IFN-λ3-GFP or IFN-λ4-GFP and used CRISPR-Cas9 gene editing to eliminate IFNLR1, the receptor used by all type III IFNs (**Figure S1**). After doxycycline (dox)-induction of these cell lines for 8, 24, and 72 hrs, we analyzed their global transcriptome by RNA-seq.

**Figure 1:**
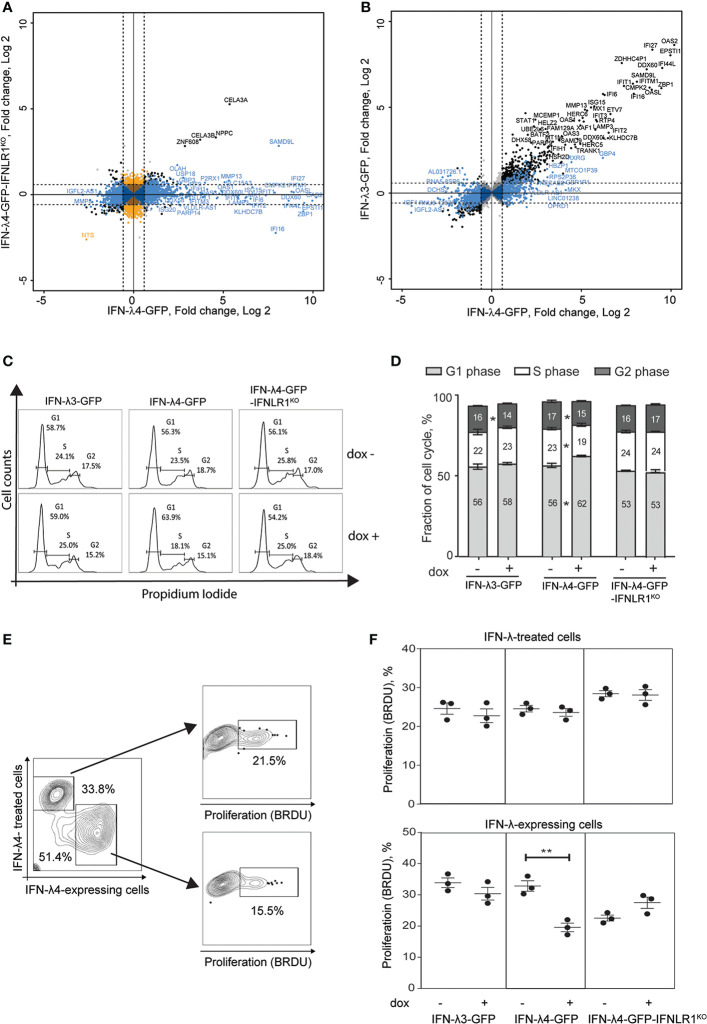
IFN-λ4 inhibits the proliferation of HepG2 cells. RNA-sequencing analysis was used to identify differentially expressed genes (DEGs, P-FDR < 0.05) in IFN-λ3-GFP, IFN-λ4-GFP and IFN-λ4-GFP-IFNLR1^KO^ HepG2 cells after 72 hrs of induction by dox, compared to controls (dox- conditions). The cutoff threshold (fold change > +/-1.5) is indicated by dotted lines. **(A)** Analysis of all DEGs (n = 3251) detected for IFN-λ4-GFP or IFN-λ4-GFP-IFNLR1^KO^ cells. In blue - DEGs (n = 2,735) specific to IFN-λ4-GFP and considered IFNLR1-dependent. In black - DEGs (n = 145) shared between both groups and considered IFNLR1-independent. In orange - DEGs (n = 371) specific to IFN-λ4-GFP-IFNLR1^KO^. **(B)** DEGs of IFN-λ4-GFP analyzed in IFN-λ3-GFP transcriptome. In black - DEGs (n = 1,506) shared in IFN-λ4-GFP and IFN-λ3-GFP and in blue - IFN-λ4-signature DEGs (n = 1,229) detected in IFN-λ4-GFP but not in IFN-λ3-GFP producing cells. Additional details are provided in **Figure S2** and **Table S1**. **(C, D)** Cell cycle analysis of cells synchronized by 24 hrs of serum starvation, treated with or without dox (0.5 µg/ml) for 72 hrs, and analyzed by flow cytometry after PI staining. The plot shows a representative picture and the percentage of cells in each phase of the cell cycle. All data are shown as mean± SEM from triplicate experiments. *P < 0.05, Student’s T-test comparing no dox to dox+ for each cell cycle stage. **(E, F)** Bromodeoxyuridine (BrdU, %) incorporation indicating cell proliferation in HepG2 cells expressing IFN-λ3-GFP, IFN-λ4-GFP and IFN-λ4-GFP-IFNLR1KO. Cells were cocultured with HepG2 cells labeled with Far Red proliferation dye, dox-induced for 72 hrs, and treated with BrdU for 3 hrs before analysis. Gates show HepG2 cells exposed to IFN-λs (IFN-λ treated cells) and HepG2 expressing IFN-λs. P-values compare corresponding dox+ *vs.* dox- HepG2 cells, **p < 0.01, Student’s T-test. Graphs represent one of three independent experiments, each in biological triplicates.

IFN-λ4 induction for 72 hrs resulted in the most extensive set of differentially expressed genes (DEGs, P-FDR < 0.05 and dox+/dox-fold change +/-≥ 1.5, n=2880); this set was used for further comparative analyses (**Figure S2** and **Table S1**). Of these genes, 2735 and 145 were classified as IFNLR1-dependent and -independent, respectively, based on their differential expression in IFN-λ4-GFP and IFN-λ4-GFP-IFNLR1^KO^ cells (**Figure 1A**).

The set of IFN-λ4-induced and IFNLR1-dependent genes (n=2735) was then compared with the expression of the same gene set in IFN-λ3-producing cells. *IFNL3* and *IFNL4* transcripts in these cell lines were induced to a similar magnitude (**Figure S3A**), although actual protein levels may differ. A set of 1506 genes was induced by IFN-λ3 or IFN-λ4 (P-FDR < 0.05), with 766 genes induced by ≥1.5 fold in both groups. As expected, ISGs were the top-induced genes activated stronger by IFN-λ4 compared to IFN-λ3 (**Figure S3B** and **Figure 1B**). The remaining 1229 (of 2735) genes were considered as IFN-λ4-specific DEGs (**Figure S2G** and **Table S1**) and enriched with genes related to cell cycle inhibition (**Figure S3C** and **Table S1**).

In line with our previous results (37), we observed G1/G0 cell cycle arrest (**Figures 1C, D** **)** and reduced proliferation (**Figure S4**) in IFN-λ4-expressing HepG2 cells. However, another study did not demonstrate the antiproliferative effect of recombinant IFN-λ4 in hepatic cells (38). To address this discrepancy, we examined proliferation in HepG2 cells expressing IFN-λ4 (and thus exposed to this recombinant protein both endogenously and exogenously), and bystander cells not producing but only exposed to secreted IFN-λ4 exogenously. We used cell-permeable dyes to discriminate IFN-λ4-expressing from IFN-λ4-exposed HepG2 cells and estimated proliferation in both populations using a flow cytometry-based proliferation assay. We observed that in contrast with IFN-λ4-expressing cells there was no inhibition of proliferation in bystander cells exposed to recombinant IFN-λ4 (**Figures 1E, F**). Interestingly, the antiproliferative effect of intracellular IFN-λ4 was also attenuated in IFN-λ4-GFP-IFNLR1^KO^ cells (**Figures 1E, F**). These results indicate that IFN-λ4 causes cell cycle arrest and reduced proliferation of hepatic cells through a mechanism that is IFNLR1-dependent and requires intracellular expression of IFN-λ4.

### IFN-λ4 Is a Misfolded Protein That Induces a Potent ER Stress Response in Hepatic Cells

The absence of antiproliferative effects in HepG2 cells exposed to IFN-λ4 (**Figures 1E, F**) suggests that paracrine IFN signaling was insufficient to inhibit cell proliferation, prompting us to search for additional intrinsic factors associated with IFN-λ4 expression. Unfolded protein response (UPR) was one of the main pathways we identified by Ingenuity Pathway Analysis (IPA) as significantly activated in IFN-λ4-expressing HepG2 cells (**Figure S5**). UPR due to endoplasmic reticulum (ER) stress has been shown to inhibit proliferation and induce apoptosis in some conditions (39, 40). UPR is induced to relieve ER stress by increasing the expression of several chaperones that aid protein folding (39, 41), and we found a more robust induction of select UPR effectors, curated from IPA, in IFN-λ4-expressing compared to IFN-λ3-expressing and IFN-λ4-IFNLR1^KO^ HepG2 cells (**Figures S6A–C**). We previously showed that infection of primary human hepatocytes (PHH) with Sendai virus (SeV) resulted in intracellular accumulation of IFN-λ4 (25), possibly causing ER stress. We observed that the expression of an ER stress maker, *DDIT3*, was significantly upregulated in PHH from donors with *vs.* without *IFNL4* genotype (**Figure S6D**), while for other ER stress markers, we observed a trend, which did not reach statistical significance, possibly due to low sample size (**Figure S6E**).

**Figure 2:**
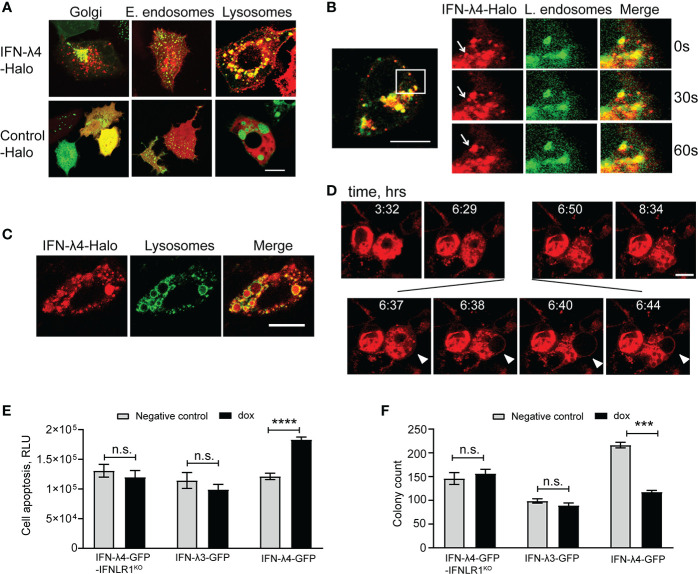
IFN-λ4 is a misfolded protein that induces ER stress. Representative confocal images of HepG2 cells transduced with a mammalian baculovirus delivery system (BacMam) of GFP-tagged proteins targeting specific organelles - lysosomes, Golgi, early and late endosomes. After transduction for 6 hrs, cells were transiently transfected with Halo-tagged constructs for IFN-λ4 or control for indicated times, stained with cell-permeant Halo-tag ligand TMR (red), and imaged. **(A)** Confocal images showing IFN-λ4 accumulation in lysosomes but not in early endosomes. **(B)** Late endosomal trafficking of IFN-λ4, with the inset showing larger magnification. **(C)** Unfolded protein response (UPR) is represented by lysosomal enlargement after protein accumulation. **(D)** Live images of IFN-λ4-expressing HepG2 cells undergoing apoptosis, characterized by membrane blebbing and cell death. Images were scanned every minute for 12 hrs. Scale bars – 10 um. **(E)** Apoptosis detection with ApoTox-Glo assays in corresponding untreated and dox-induced cells for indicated time points. RLU, relative luminescence units. **(F)** Graph showing counts from colony formation assay for HepG2 cells expressing IFN-λ3, IFN-λ4 or IFNLR1 KO grown in 6-well plates with or without dox for 13 days. Cell colonies were stained with crystal violet and counted with ImageJ software. The graph represents the number of colonies as a percentage of initial plated counts. n.s., not significant, ***p < 0.001, ****p < 0.0001, Student’s T-test.

The induction of ER stress (**Figures S6A–C**) and intracellular accumulation of IFN-λ4 (**Figures 1E, F**) (25, 37) suggested that IFN-λ4 is an inefficiently folded protein that induces the misfolded protein ER stress response (40, 42). ER stress is mitigated by targeting misfolded proteins to lysosomes for degradation (39, 42). Live imaging in HepG2 cells showed that IFN-λ4 was accumulated in lysosomes (**Figure 2A**) *via* late endosome trafficking (**Figure 2B** and **Video S1**) but was excluded from early recycling endosomes (**Figure 2A**). Continued accumulation of IFN-λ4 led to lysosomal enlargement (**Figure 2C**), followed by membrane blebbing and cell death, implicating apoptosis (**Figure 2D** and **Video S2**). We confirmed apoptosis in IFN-λ4-expressing cells using a biochemical assay for caspase activity (**Figure 2E**) and cell viability (**Figure 2F**). Prolonged ER stress causes apoptosis *via* the activity of the UPR-induced transcription factor DDIT3 (43), downregulating the expression of anti-apoptotic factors, including BCL2 (44), which we also observed (**Figure S6A**). These results suggest that prolonged ER stress due to the continued endogenous production of misfolded IFN-λ4 could be contributing to apoptosis of hepatic cells.

### IFN-λ4 Induces ER Stress and Inhibits Proliferation in Hepatic Stellate Cells

**Figure 3:**
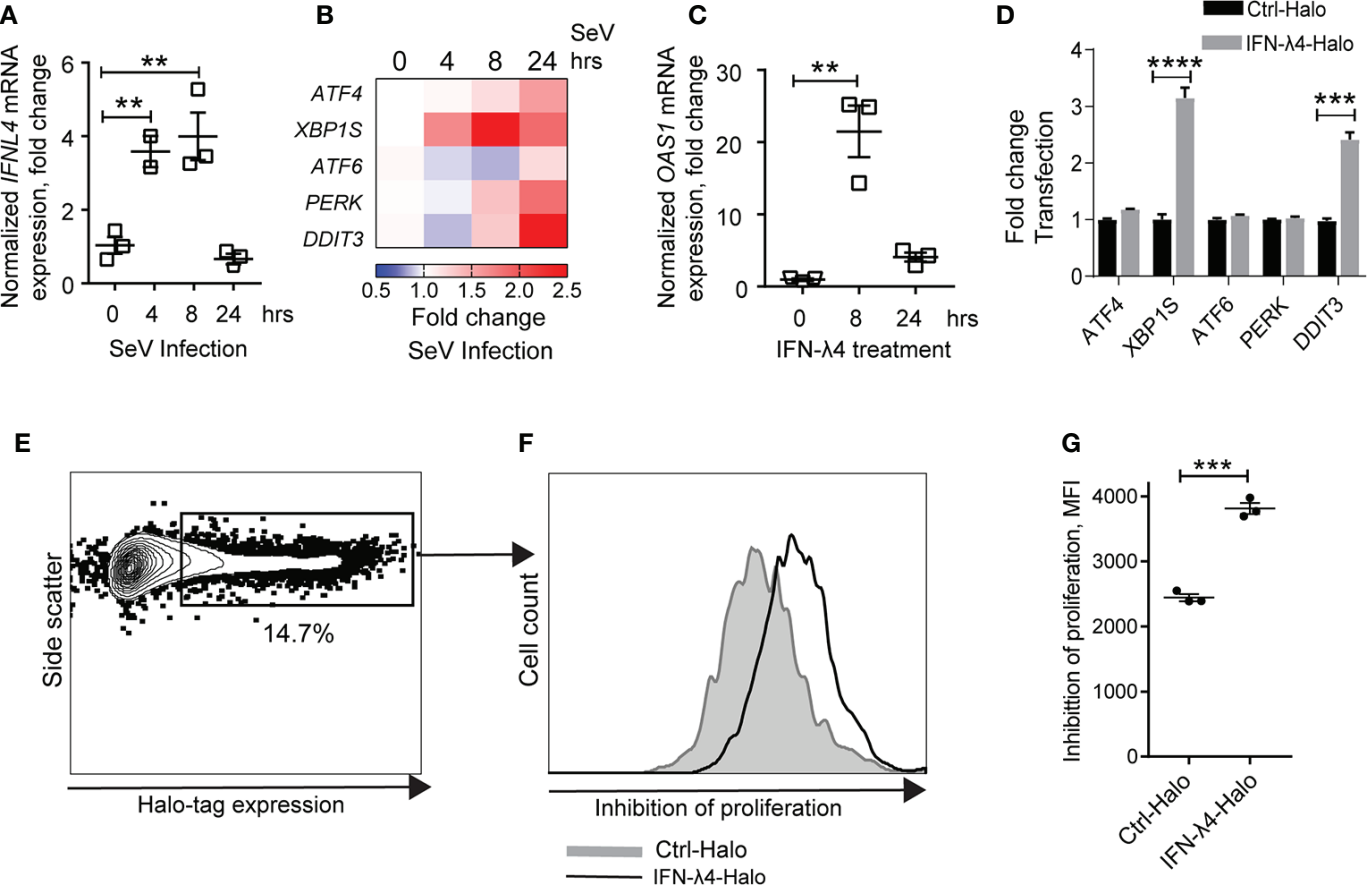
IFN-λ4 inhibits the proliferation of hepatic stellate cells (HSCs). qRT-PCR expression analysis showing **(A)** *IFNL4* expression after SeV infection of primary HSCs and **(B)** induction of UPR markers**. (C)** qRT-PCR showing induction of *OAS1* after treatment of primary HSCs with recombinant IFN-λ4 (50ng/ml). **(D)** qRT-PCR showing induction of UPR markers in LX-2 hepatic stellate cell line transiently transfected for 24 hrs with Halo-tagged constructs for IFN-λ4 or control. **(E–G)** Proliferation analysis in LX-2 cells transiently transfected with Halo-tagged constructs for IFN-λ4 or control, labeled with Far Red proliferation dye and analyzed by flow cytometry after 72 hrs. **(E)** Gating for Halo-tag expression. **(F)** Histogram representing the proliferation of gated cells, comparing cells expressing Halo-tagged IFN-λ4 *vs.* control. **(G)** Graph representing the geometric mean expression of Far Red proliferation dye with higher values indicating reduced proliferation. **p < 0.01, ***p < 0.001, ****p < 0.0001, Student’s T-test.

Activated hepatic stellate cells (HSCs) are key drivers of liver fibrosis during HCV infection (45). While HCV is not known to replicate in HSCs, type-III IFNs can be produced in HSCs in response to poly I:C, while another study showed that HCV binds HSCs *via* CD81 where it upregulates MMP2 (46). Thus, we examined whether IFN-λ4 can induce ER stress not only in hepatocytes but also in HSCs. We infected primary HSCs with SeV to induce IFNs and observed significant induction both of *IFNL4* (**Figure 3A**) and ER stress genes (**Figure 3B**). The induction of *OAS1*, an ISG, in response to IFN-λ4 treatment indicated the presence of the functional type III signaling machinery, including IFNLR1, in primary HSCs (**Figure 3C**). When we overexpressed IFN-λ4 in LX2 (a human stellate cell line), similarly to hepatocytes, we observed upregulation of ER stress genes and inhibition of cell proliferation (**Figures 3D–G**). These results suggest that decreased proliferation of activated HSCs, which has been shown to contribute to reduced cirrhosis (47), could partly explain the association of *IFNL4* genotype with reduced cirrhosis in patients with HCV.

### IFN-λ4 Associated ER Stress Induces *VLDLR* Expression

**Figure 4:**
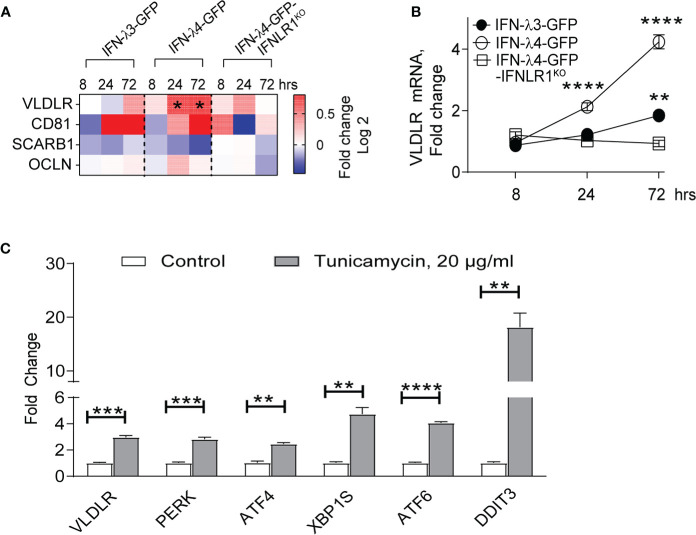
IFN-λ4 upregulates *VLDLR* expression. **(A)** Differential expression of HCV receptors identified by RNA-seq analysis (**Table S1**) comparing corresponding dox+ and dox- HepG2 cells induced for indicated times points and presented as fold change (Log2). * represents significant (p<0.05) induction above 1.5 fold. **(B)** qRT-PCR analysis of *VLDLR* after IFN-λ4 induction in corresponding HepG2 cells. Mean +/- SEM **(C)** qRT-PCR analysis of *VLDLR*, as well as select UPR-associated genes in HepG2 cells following tunicamycin treatment for 24 hours. Each gene expression was normalized by the expression of endogenous controls (*GAPDH* and *ACTB*) and by expression in corresponding untreated groups. **P < 0.01, ***P < 0.001, ****P < 0.0001, Student’s T-test.

Primary receptors for HCV include occludin (OCLN) (48), CD81, scavenger receptor class B type 1 (SCARB1), low-density lipoprotein receptor (LDLR), and surface glycosaminoglycans (49). Very-low-density lipoprotein receptor (VLDLR) was recently identified as an additional receptor for HCV (50). Since VLDLR is induced in response to ER stress in some conditions (51), we tested if IFN-λ4-induced ER stress could be contributing to the upregulation of *VLDLR* expression. RNA-seq data showed significant upregulation of *VLDLR*, while other receptors (*OCLN*, *CD81* and *SCARB1*) were not induced in response to IFN-λ4 expression in HepG2 cells (**Figure 4A**). Analysis with gene-specific expression assays using independent biological replicates of HepG2 cells validated the induction of *VLDLR* by IFN-λ4 (**Figure 4B**). We then tested whether ER stress could lead to upregulation of *VLDLR.* Indeed, in normal HepG2 cells, tunicamycin treatment led to significant upregulation of *VLDLR* (by 3-fold, **Figure 4C**). These results suggest that IFN-λ4 could be increasing the expression of *VLDLR* in hepatic cells by inducing ER stress, thus, promoting and sustaining HCV entry.

## Discussion

Genetic variants within the *IFNL3*/*IFNL4* region have been associated with increased risk of chronic HCV but reduced fibrosis. Reconciling these opposing phenotypes has been further complicated by strong linkage disequilibrium in this locus (6), nominating several *IFNL3* and *IFNL4* variants as potentially contributing to these associations. We used *IFNL4* genotype as a representative marker in this region to explore genetic associations in large sets of HCV patients from Japan and Taiwan. We observed a moderately increased risk of progression to HCC in HCV patients with *IFNL4* genotype in the general population, but this risk was eliminated in patients achieving viral clearance, explaining inconsistencies in previous reports on HCC risk (10–15). Thus, our results support the role of IFN-λ4 in HCV risk but not in progression to HCC once HCV is cleared.

We also observed that HCV patients with *IFNL4* genotype were less likely to manifest liver cirrhosis, in line with the previous studies (16, 17, 34). Our data suggest that reduced fibrosis/cirrhosis could be due to ER stress induced by intracellular accumulation of IFN-λ4, but not IFN-λ3. Specifically, we observed that inducible expression of IFN-λ4 was associated with cell cycle arrest, apoptosis, and decreased cell proliferation. In addition to primary hepatocytes, HSCs could be critical for antiproliferative effects of IFN-λ4. Viral hepatitis is characterized by repeated cycles of hepatic cell death and regeneration. Breakdown in the formation of the extracellular matrix in combination with continuous proliferative pressure leads to the development of fibrotic tissue (18, 52–54). This mechanism is mediated by HSCs, which differentiate into myofibroblasts and accumulate in fibrotic tissue (54, 55). As a result, signaling pathways that lead to cell cycle arrest in HSCs inhibit the development of fibrosis and cirrhosis (47, 54). Indeed, we observed that IFN-λ4-induced ER stress coupled with IFN signaling reduced proliferation in HSCs. Although the role of ER stress in liver homeostasis is complex, with conflicting findings emerging from mouse knockout studies (53), induction of ER stress as an adaptive process could be beneficial in other inflammatory conditions of the liver, such as non-alcohol fatty liver disease (NAFLD) (53).

We were somewhat surprised that IFNLR1 knockout attenuated the ER-stress response, suggesting a role for type-III IFN signaling in the antiproliferative effect, although IFN-λ3-GFP expression did not induce ER-stress. We propose that IFN-λ4 associated UPR coupled with type-III IFN signaling leads to significant ER-stress and antiproliferative effects. Some studies have shown that type-I IFNs can induce ER-stress in certain cancer cells (56–58). Although acting through different receptors, type-I and type-III IFNs induce a similar set of ISGs, supporting the idea that in the right context, type-III IFNs could induce ER-stress. This may be the case for IFN-λ4, where ER-stress could be induced by type-III IFN signaling coupled with UPR, while neither mechanism may be sufficient to induce ER stress on its own.

Our experimental model utilized hepatic cell lines designed to examine the specific effects of IFN-λ4 or the closely related IFN-λ3 in mediating antiviral effects. This approach enabled us to identify novel phenotypes associated with IFN-λ4 expression. Importantly, in virally infected PHH, where we previously demonstrated intracellular accumulation of IFN-λ4 (25), *IFNL4* genotype was also associated with increased ER stress, supporting the role of endogenously expressed IFN-λ4 in contributing to increased ER stress during viral infections.

Several mechanisms have been proposed for the association of *IFNL4* genotype with HCV persistence. We and others have demonstrated an association of *IFNL4* genotype with increased ISG induction and enhanced negative regulation of IFN responses (7, 25). Here, we describe a potentially novel mechanism contributing to the association of *IFNL4* with chronic HCV *via* the upregulation of VLDLR, a putative entry receptor for HCV (50). LDLs and VLDLs can shield HCV from antibody neutralization (59), and their circulating levels can be reduced by increased expression of VLDLR in the liver (60). Interestingly, *IFNL4* genotype has been associated with lower circulating levels of LDL in HCV (61), which may be linked to increased VLDLR expression. ER stress-induced VLDLR expression appears to be dependent on protein kinase RNA‐like ER kinase–activating transcription factor 4 (PERK) signaling pathway (51), which was also induced by IFN-λ4 overexpression in our models.

In conclusion, we present genetic and functional results suggesting a role for IFN-λ4 in antiproliferative mechanisms *via* intracellular accumulation and induction of ER stress, with implications for the development of HCV, cirrhosis, and HCC. We acknowledge the limitations of our study, including the lack of animal models complicated by the absence of *IFNL4* in the mouse genome. Further dissecting the role of IFN-λ4 in the context of HCV infection and developing clinically relevant applications will require replicating these findings in comparable cohorts of patients and controlling for HCV treatments and outcomes.

## Data Availability Statement

The RNA-seq dataset for 108 samples (54 individual samples in duplicates) has been deposited to NCBI Gene Expression Omnibus (GEO) with an accession number GSE145038 and is accessible through the link https://www.ncbi.nlm.nih.gov/geo/query/acc.cgi?acc=GSE145038.

## Ethics Statement

The study was conducted in accordance with the ethical principles stated in the Declaration of Helsinki and was approved by the Ethics Committee of the National Taiwan University Hospital, Kaohsiung Medical University Hospital, China Medical University Hospital, Chang Gung Memorial Hospital in Kaohsiung and Linkou, and Academia Sinica. The project was approved by the ethical committees of the University of Tokyo. The patients/participants provided their written informed consent to participate in this study.

## Author Contributions

OO and LP-O: Study concept and design. OO, FW, M-HL, AO, CT, JV, S-FL, CS, Y-HH, C-YS, AB, ZH, and KM: Acquisition of data. OO, FW, M-HL, OF-V, AO, CT, JV, SF-L, CS, Y-HH, C-YS, AB, TO’B, ZH, KM, and LP-O: Analysis and interpretation of data. OO and LP-O: Drafting of the manuscript. OO, FW, M-HL, OF-V, TO’B, and LP-O: Critical revision of the manuscript for important intellectual content. OO, FW, M-HL, OF-V, and LP-O: Statistical analysis. LP-O, M-HL: Obtained funding, technical, or material support. LP-O: Study supervision. All authors contributed to the article and approved the submitted version.

## Funding

This study was supported by the Intramural Research Program of the Division of Cancer Epidemiology and Genetics, US National Cancer Institute, research grants from the Ministry of Science and Technology, Taipei, Taiwan (105-2628-B-010-003-MY4 and 107-2314-B-010-004-MY2). Funders had no role in study design; collection, management, analysis, and interpretation of the data; preparation, review, or approval of the manuscript, and decision to submit the manuscript for publication.

## Conflict of Interest

TO’B and LP-O are co-inventors on IFN-λ4-related patents issued to NCI/NIH and receive royalties for antibodies for IFN-λ4 detection.

The remaining authors declare that the research was conducted in the absence of any commercial or financial relationships that could be construed as a potential conflict of interest.

## Publisher’s Note

All claims expressed in this article are solely those of the authors and do not necessarily represent those of their affiliated organizations, or those of the publisher, the editors and the reviewers. Any product that may be evaluated in this article, or claim that may be made by its manufacturer, is not guaranteed or endorsed by the publisher.
