# Supplementary material for: Intracellular Accumulation of IFN-λ4 Induces ER Stress and Results in Anti-Cirrhotic but Pro-HCV Effects
Source: Front Immunol. 2021 Aug 23;12:692263. doi: 10.3389/fimmu.2021.692263 (PMC8419317; doi:10.3389/fimmu.2021.692263)
Supplement: Supplementary file 1 [file DataSheet_1.docx]

**Intracellular accumulation of IFN-λ4 induces ER stress and results in anti-cirrhotic but pro-HCV effects**

**Onabajo, Wang, Lee et al**

**SUPPLEMENTARY MATERIALS**

**Supplementary Tables**

**Table S1.** List of DEGs significantly induced by IFN-λ4-GFP, separate Excel file

**Table S2.** Primers and expression assays used in the study

**Supplementary Figures**

**Figure S1.** Generation of the IFN-λ4-GFP-IFNLR1^KO^ HepG2 cell line using CRISPR/Cas9 genome editing

**Figure S2.** Outline of RNA-seq data analysis in HepG2 cells

**Figure S3.** IFNLR1-dependent pathways affected by IFN-λ4-GFP expression in HepG2 cells.

**Figure S4.** IFN-λ4 expression significantly inhibits proliferation in HepG2 cells

**Figure S5.** Pathways for IFNLR1-dependent DEGs significantly affected by IFN-λ4-GFP expression in HepG2 cells

**Figure S6.** IFN-λ4 expression induces signaling pathways related to unfolded protein response (UPR).

**Figure S7.** Inhibition of proliferation by IFN-λ4-GFP demonstrated by colony formation assay

**Supplementary Videos**

**Video S1**. Trafficking of IFN-λ4 in late endosomes in HepG2 cells

**Video S2**. Apoptosis of HepG2 cells expressing IFN-λ4

**SUPPLEMENTARY TABLES**

**Table S2.** **Primers and expression assays used in the study**

|  | Primer or gene names | Primer sequence (5’-3’) or gene assays |
| --- | --- | --- |
| ***IFNLR1*** | Exon3-F | CAAAGCTAGGGAGGGACAGGG |
| **Primers** | Exon3-R | CTGACAGGCTACCAGACATG |
|  | Sequencing primer | GTCATCAACCCGCTCCAAGG |
| **TaqMan expression assays** (Thermo Fisher Scientific) | | |
|  | *GAPDH* | Hs02786624_g1 |
|  | *ACTB* | 4326315E |
|  | *MX1* | Hs00895608_m1 |
|  | *ISG15* | ISG15 Hs01921425_s1 |
|  | *OAS1* | Hs00973637_m1 |
|  | *CXCL10* | [Hs00171042_m1](https://www.thermofisher.com/taqman-gene-expression/product/Hs00171042_m1?CID=&ICID=&subtype=) |
|  | *PD-L1* | [Hs01125301_m1](https://www.thermofisher.com/taqman-gene-expression/product/Hs01125301_m1?CID=&ICID=&subtype=) |
|  | *PERK* | [Hs00984005_m1](https://www.thermofisher.com/taqman-gene-expression/product/Hs00984005_m1?CID=&ICID=&subtype=) |
|  | *ATF4* | [Hs00909569_g1](https://www.thermofisher.com/taqman-gene-expression/product/Hs00909569_g1?CID=&ICID=&subtype=) |
|  | *ATF6* | [Hs00232586_m1](https://www.thermofisher.com/taqman-gene-expression/product/Hs00232586_m1?CID=&ICID=&subtype=) |
|  | *DDIT3* | [Hs00358796_g1](https://www.thermofisher.com/taqman-gene-expression/product/Hs00358796_g1?CID=&ICID=&subtype=) |
|  | *VLDLR* | [Hs01045914_g1](https://www.thermofisher.com/taqman-gene-expression/product/Hs01045914_g1?CID=&ICID=&subtype=)[Hs03309901_g1](https://www.thermofisher.com/taqman-gene-expression/product/Hs03309901_g1?CID=&ICID=&subtype=) |
| **Custom TaqMan expression assay** (Thermo Fisher Scientific) | | |
| *XBP1S* | Forward primer | GCTGAGTCCGCAGCAGGT |
|  | Probe | CCAACAGGATATCAGACTCTGAATCT |
|  | Reverse primer | CAGAACATCTCCCCATGGA |

**SUPPLEMENTARY FIGURES**

**
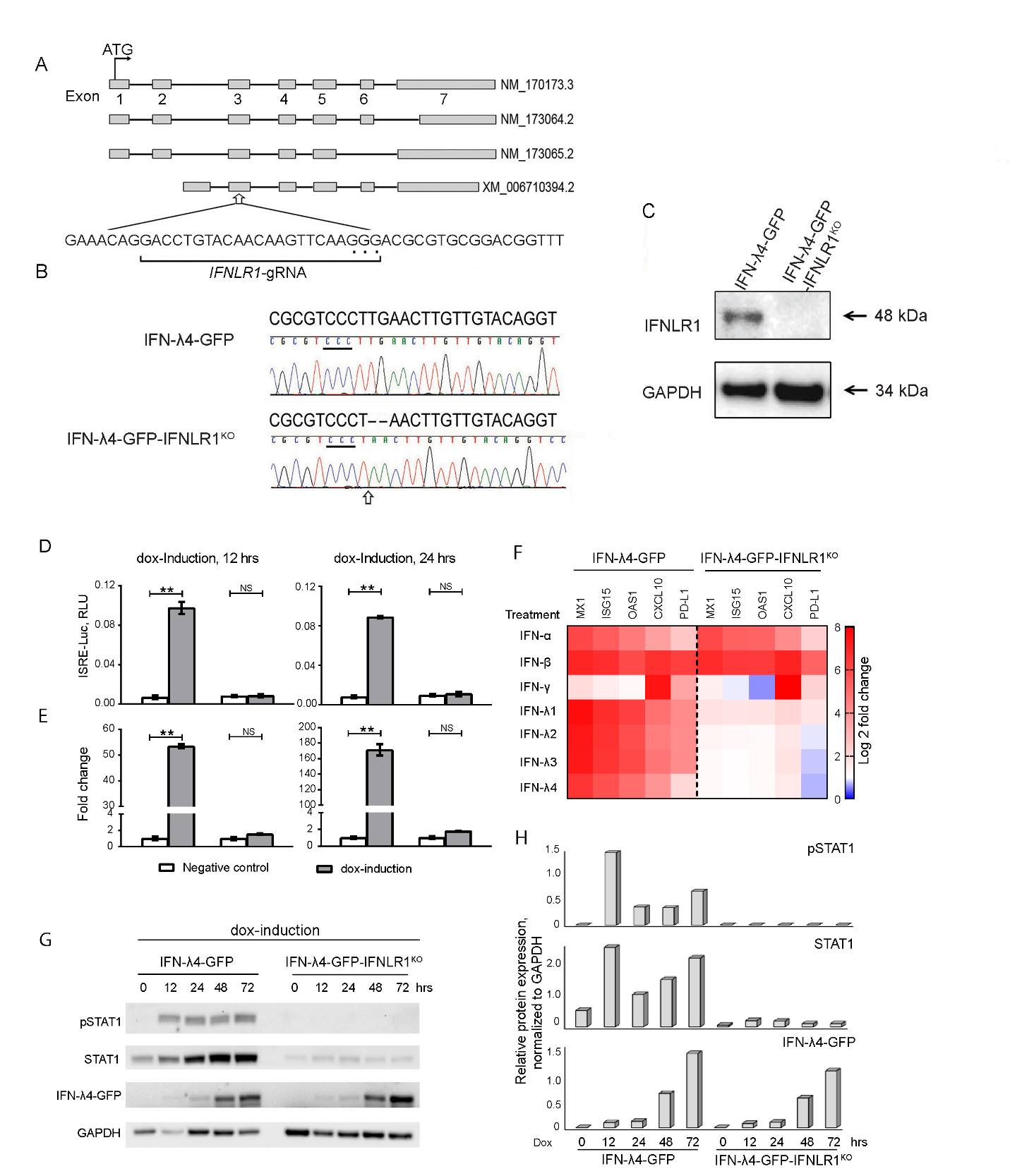
**

**Fig. S1. Generation of the IFN-λ4-GFP-IFNLR1^KO^ HepG2 cell line using CRISPR/Cas9 genome editing.**

All type III IFNs signal via a heterodimeric receptor complex composed of the ubiquitously expressed interleukin 10 receptor 2 (IL10R2) that is shared by many cytokines, and the interferon lambda receptor 1 (IFNLR1), which is primarily expressed in epithelial and hepatic cells and serves only type III IFNs. Both receptors are expressed in HepG2, a hepatoma cell line, which responds to the signaling of type III IFNs. The HepG2-IFNLR1^KO^ cell line was created by eliminating *IFNLR1* in HepG2 cells by CRISPR-Cas9 genome editing. **(A)** Genomic organization of the *IFNLR1* transcripts. Arrow indicates the region within the common exon 3 targeted by all six *IFNLR1*-gRNAs tested, with exon numbering based on the NM_170173.3 transcript. Target sites and protospacer adjacent motif (PAM) sequences for the most efficient *IFNLR1*-gRNA are marked by lines and dots, respectively. **(B)** Sequence alignment of the target region of *IFNLR1* exon 3 in the IFN-λ4-GFP and IFN-λ4-GFP-IFNLR1^KO^ HepG2 cell lines shows a 2 bp deletion that is expected to eliminate the IFNLR1 protein. **(C)** Complete elimination of the IFNLR1 protein is validated by Western blotting of cell lysates, with GAPDH used as a loading control. The top ten predicted off-target sites were tested by sequencing of the genomic DNA of IFN-λ4-GFP-IFNLR1^KO^ cells and no off-target mutations were detected (data not shown). **(D)** IFN-λ4-GFP and IFN-λ4-GFP-IFNLR1^KO^ HepG2 cells were transiently transfected in 96-well plates with the Cignal ISRE-Luc reporter plasmid and dox-induced for 12 or 24 hrs; uninduced cells were used as negative controls. Activation of the JAK/STAT signaling was evaluated by the ability to induce the ISRE-Luc reporter, measured in RLU (relative luciferase units), all based on biological triplicates per condition. **(E)** qRT-PCR expression analysis of *ISG15* with *ACTB* used as an endogenous control; the results are normalized to the control group, based on biological duplicates. **(F)** IFN-λ4-GFP and IFN-λ4-GFP-IFNLR1^KO^ cells were treated in 4 biological replicates with recombinant human proteins - IFNα (0.5 ng/ml), IFNβ (0.5 ng/ml), IFNγ (1 ng/ml), IFN-λ1 (5 ng/ml), IFN-λ2 (60 ng/ml), IFN-λ3 (20 ng/ml) and IFN-λ4 (50 ng/ml) for 8 hrs, with an untreated group used as control. Expression of *MX1*, *ISG15*, *OAS1*, *CXCL10,* and *PD-L1* was analyzed by qRT-PCR in 4 technical replicates. Heatmap shows the expression of each gene analyzed in ddCt (log2 fold change) values between treated and control groups. **(G)** Western blotting of pSTAT1, STAT1, and IFN-λ4-GFP expression in whole-cell lysates of corresponding cells, with GAPDH expression used as a loading control. **(H)** Results of Image J quantitative analysis of Western blots of pSTAT1, STAT1, and IFN-λ4-GFP expression (from panel **G**), all normalized to GAPDH. As expected, type III IFN signaling in the IFN-λ4-GFP-IFNLR1^KO^ was completely abrogated without affecting signaling of other IFNs that do not signal through IFNLR1. Error bars – SEM; ** - *P* < 0.01; NS – not significant based on two-sided Student’s T-test.


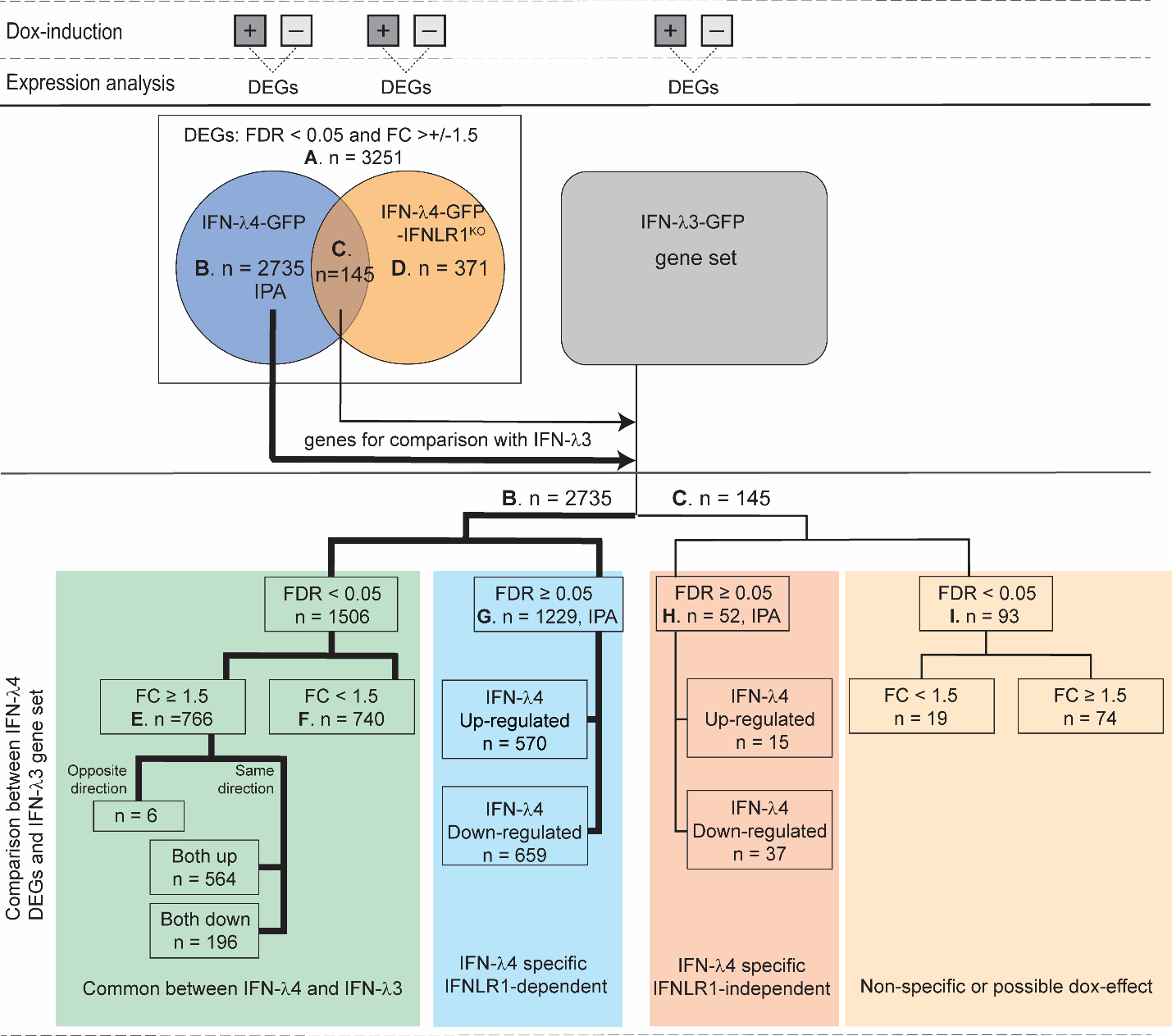


**Fig. S2. Outline of RNA-seq analysis in HepG2 cells**.

RNA-sequencing was performed using total RNA from IFN-λ4-GFP, IFN-λ4-GFP-IFNLR1^KO,^ and IFN-λ3-GFP HepG2 cells in biological triplicates. Differentially expressed genes (DEGs, FDR<0.05, +/- ≥1.5-fold change (FC)) were identified by comparing corresponding dox+ and dox- cells after 72 hrs of induction. **(A)** All DEGs (n=3,251) induced by IFN-λ4-GFP; **(B)** DEGs (n=2,735) induced by IFN-λ4-GFP and IFNLR1-dependent; **(C)** DEGs (n=145) induced by IFN-λ4-GFP and IFNLR1-independent; **(D)** DEGs (n=371) induced only in IFN-λ4-GFP-IFNLR1^KO^. DEGs unique to IFN-λ4-GFP (thick line) and those common to both IFN-λ4-GFP and IFN-λ4-GFP-IFNLR1^KO^ (thin line) were analyzed in the transcriptome of IFN-λ3-GFP cells; **(E)** DEGs (n=766) also significant in IFN-λ3-GFP (FDR<0.05, ≥1.5 FC); **(F)** DEGs (n=740) also significant (FDR<0.05) but with <1.5 FC **(G)** DEGs (n=1,229) considered IFN-λ4-GFP-specific and IFNLR1-dependent; **(H)** DEGs (n=52) considered IFN-λ4-GFP-specific and IFNLR1-independent; **(I)** DEGs (n=93) non-specifically induced in all 3 cell lines. IPA – sets of genes used for Ingenuity Pathway; additional details are provided in **Table S1.**


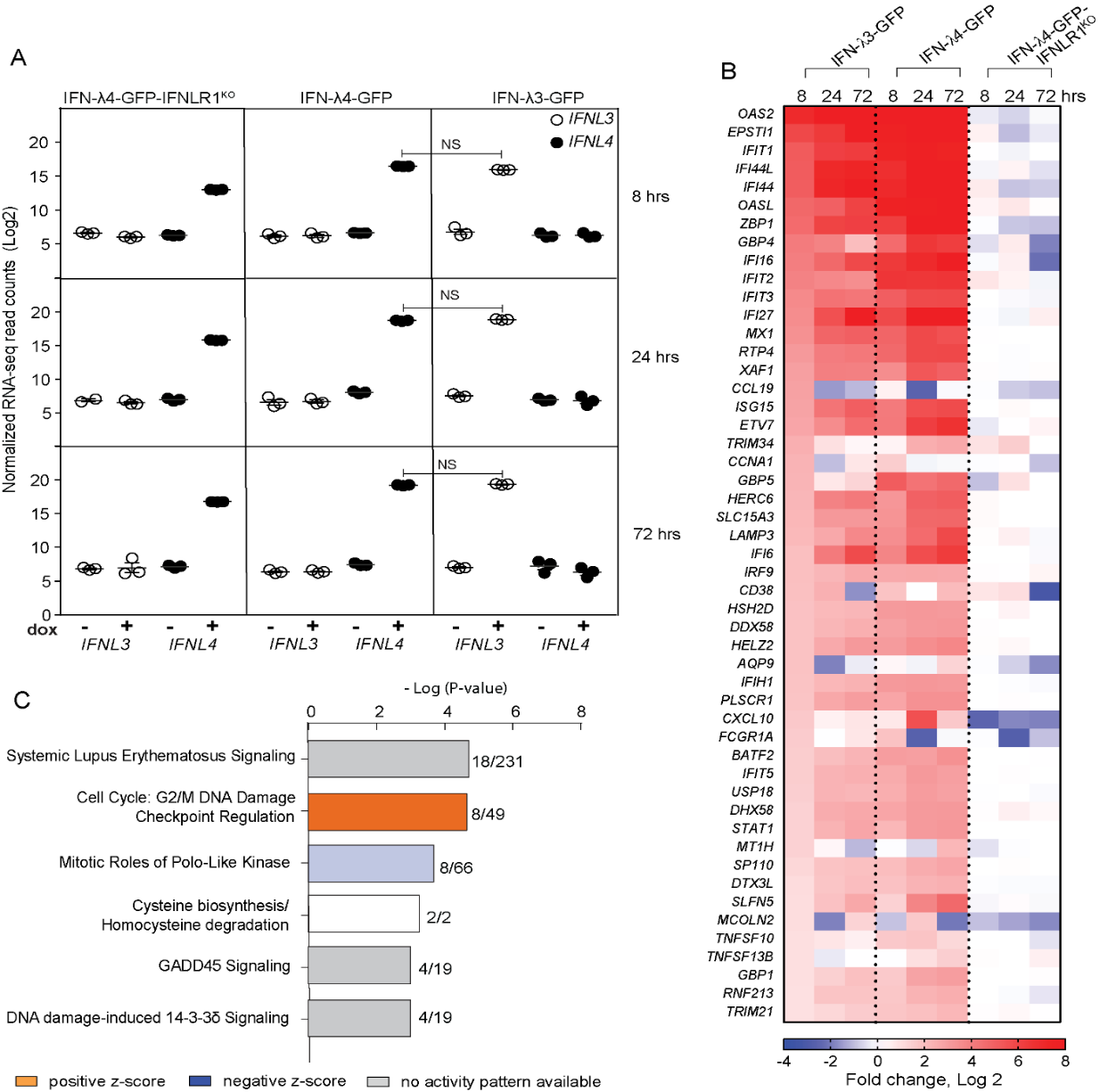


**Fig. S3.** **IFNLR1-dependent pathways affected by IFN-λ4-GFP expression in HepG2 cells.**

**(A)** Normalized counts of *IFNL3* and *IFNL4* RNA-seq reads (Log2) in corresponding cell lines with/without dox induction for indicated time points. *IFNL3* and *IFNL4* were induced specifically and to a similar magnitude. Shown - individual and mean values for biological triplicates. NS - not statistically significant based on two-sided Student’s T-test. **(B)** Differentially expressed genes (DEGs) were identified by RNA-seq analysis in IFN-λ3-GFP, IFN-λ4-GFP or IFN-λ4-GFP-IFNLR1^KO^ HepG2 cells, comparing corresponding dox+/dox-cells. Heatmap shows 50 most significantly induced IFNLR1-dependent ISGs presented as fold change (Log 2) at indicated time points; additional details are provided in **Table S1.** **(C)** Ingenuity Pathway Analysis (IPA) of 1,229 DEGs (**Fig. S2G**) in IFN-λ4-GFP HepG2 cells after 72 hrs of dox induction (P-FDR < 0.05, fold change (Log2) > +/-1.5). P-values (-log) represent the significance of gene set enrichment in each pathway. The category with a positive z-score includes genes involved in cell cycle: G2M DNA damage checkpoint regulation. Specific genes can be found in **Table S1**.

**
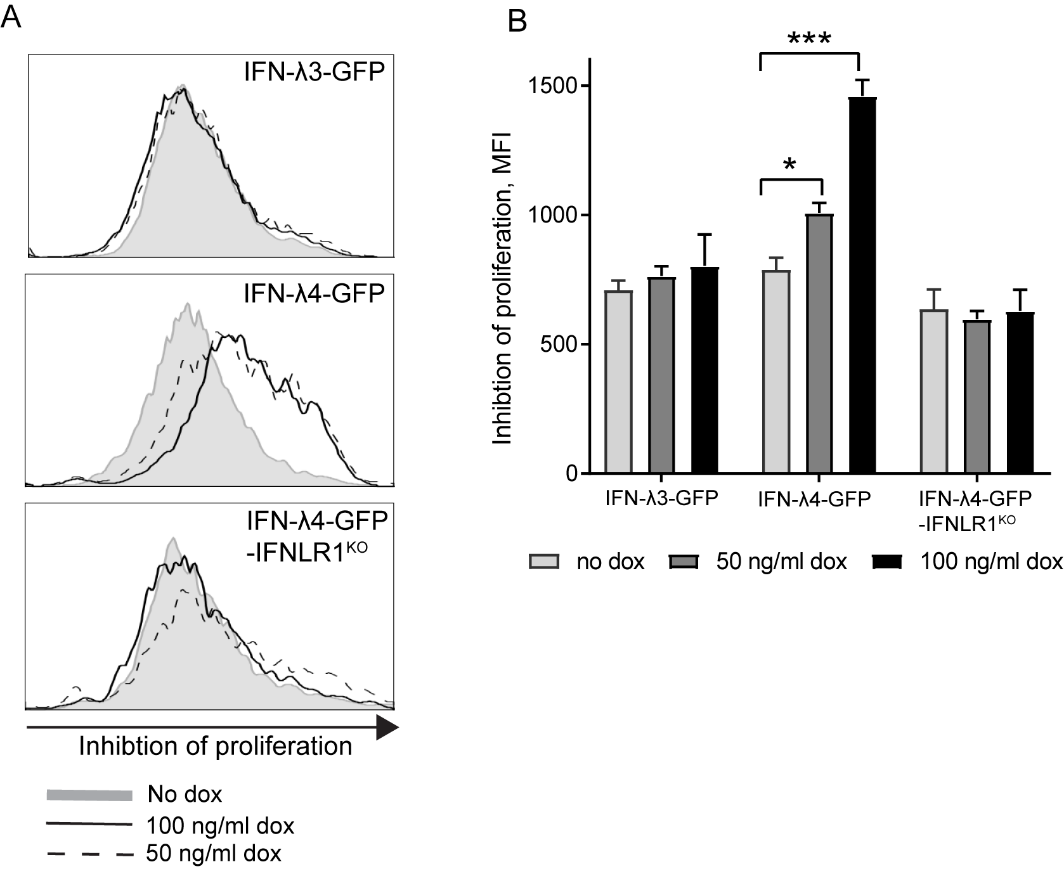
**

**Fig. S4.** **IFN-λ4 expression significantly inhibits proliferation in HepG2 cells.**

**(A)** IFN-λ3-GFP, IFN-λ4-GFP and IFN-λ4-GFP-IFNLR1^KO^ HepG2 cells were labeled with the Far Red dye to monitor cell proliferation. The cells were dox-induced for 5 days at indicated concentrations and proliferation was assessed by flow cytometry, with results presented as histograms. **(B)** Higher Far Red mean fluorescence intensity values indicate proliferation inhibition. Shown – one representative of three independent experiments. Error bars – SEM, based on 3 biological replicates. P-values are for comparisons between corresponding dox+ and dox- cells using the two-sided Student’s T-test. * p<0.05, *** p<0.001.

**
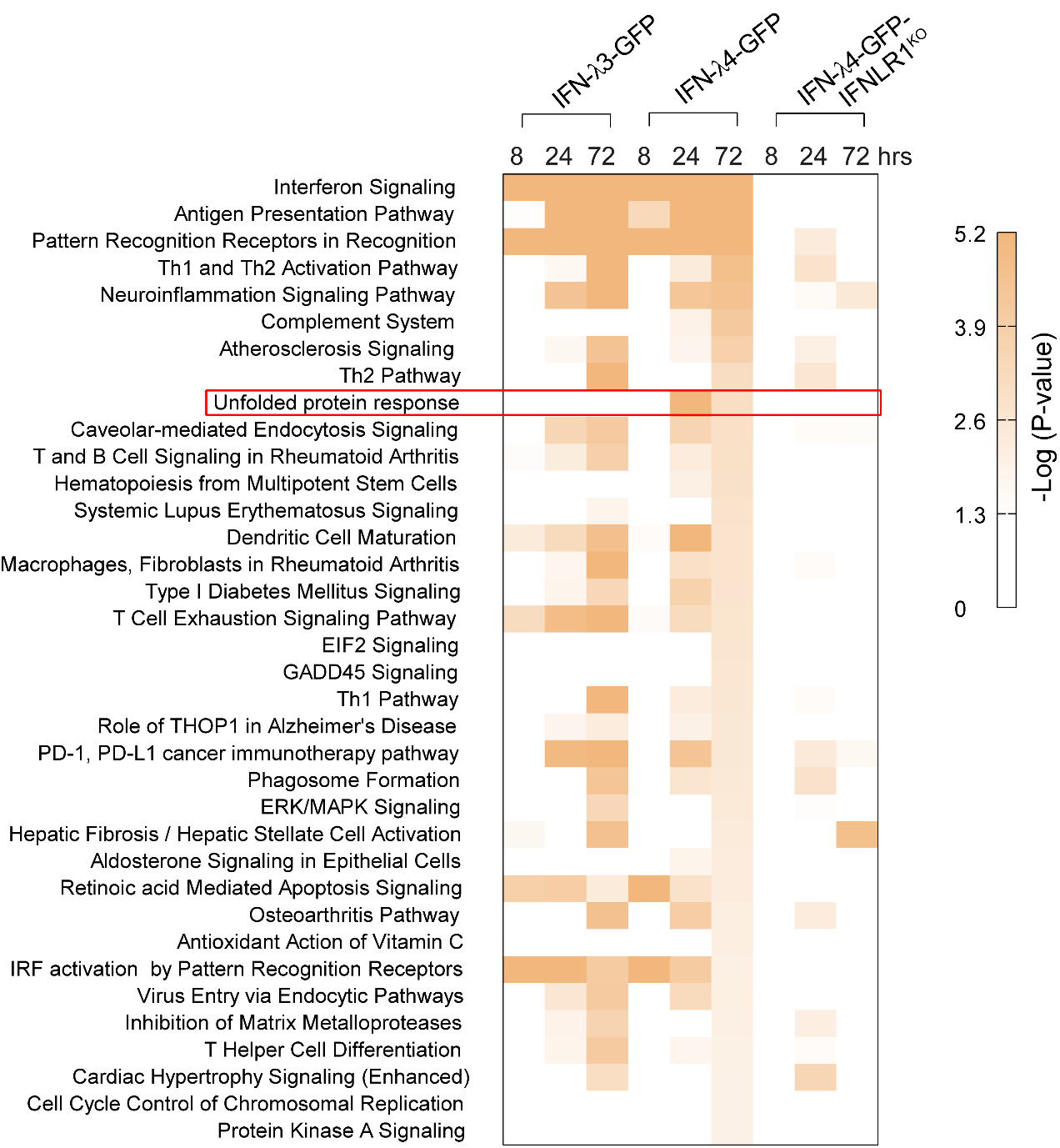
**

**Fig. S5. Pathways for IFNLR1-dependent DEGs significantly affected by IFN-λ4-GFP expression in HepG2 cells.**

Ingenuity Pathway Analysis (IPA) of differentially expressed genes (DEGs, P-FDR < 0.05, fold change > +/-1.5) identified by RNA-seq in IFN-λ3-GFP, IFN-λ4-GFP and IFN-λ4-GFP-IFNLR1^KO^ HepG2 cells comparing corresponding dox+ and dox- cells after 72 hrs of induction. All IFNLR1-dependent DEGs of IFN-λ4-GFP (n=2735, **Fig. S2B**) were used as input. Enriched pathways identified in IFN-λ4-GFP cells (P < 0.01) were plotted in a heatmap for all 3 cell lines with intensity (brown) representing (-log) P-value of the significance level of gene set enrichment. Unfolded protein response (UPR) pathway is marked as uniquely induced by IFN-λ4-GFP and not IFN-λ3-GFP.

**
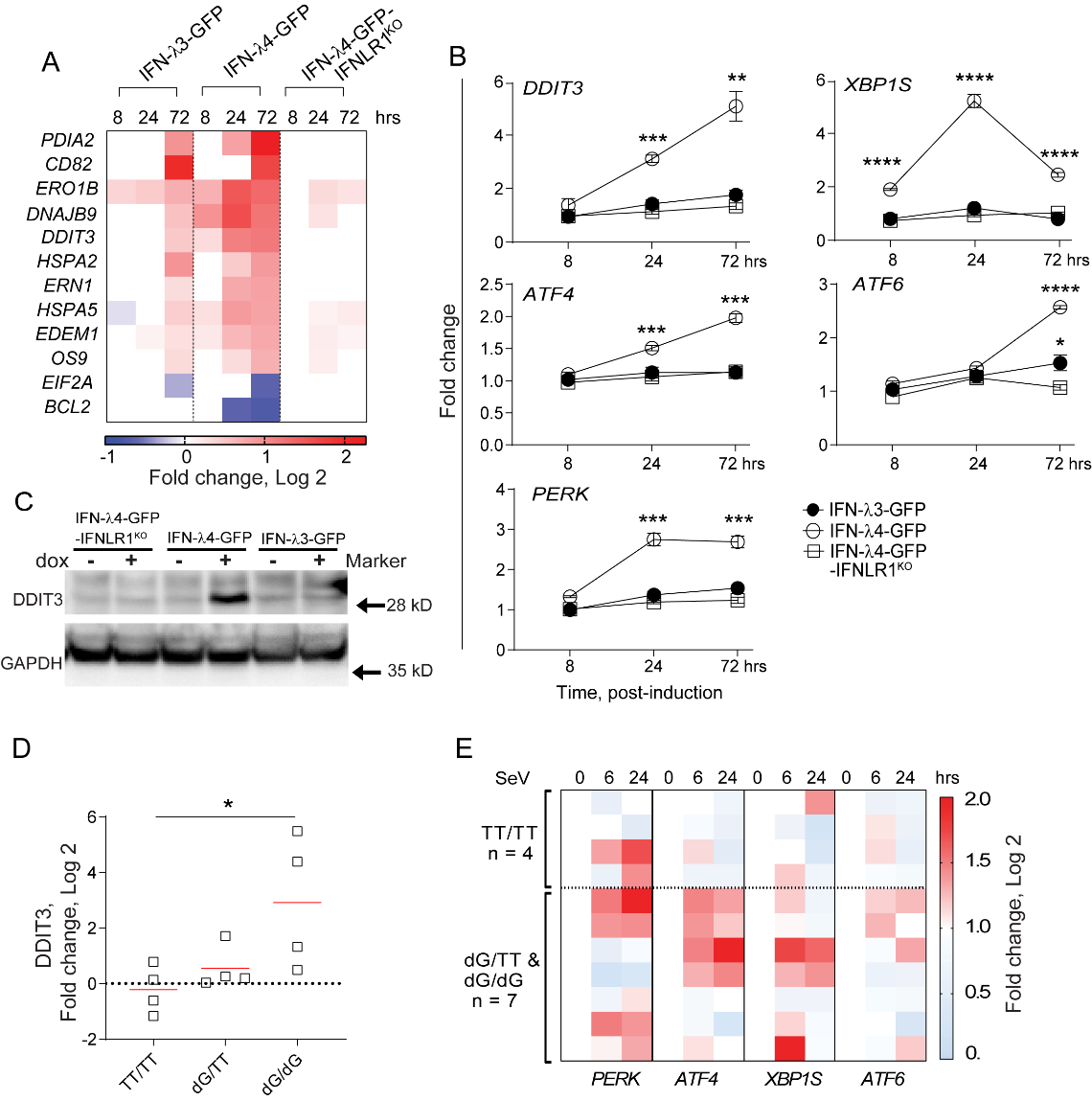
**

**Fig. S6. IFN-λ4 expression induces signaling pathways related to unfolded protein response (UPR).**

**(A)** RNA-seq data of significant differentially expressed UPR-related genes, curated from IPA, in HepG2 cells after dox-induction for indicated time points. **(B)** Graphs showing induction of several UPR-related genes based on qRT-PCR analysis in an independent set of HepG2 samples, comparing dox+ vs dox- conditions. **(C)** Western blot showing DDIT3 protein expression comparing corresponding dox+ and dox- HepG2 cells induced for 72 hrs. GAPDH is used as a loading control. **(D-E)** Endoplasmic reticulum (ER) stress is evaluated by the expression of select unfolded protein response (UPR) genes in PHH infected with SeV (7.5 × 10^5^ chicken embryo infectious dose 50 [CEID50] per milliliter) for 24 hrs. Expression was measured with qRT-PCR assays and normalized to an endogenous control (*GAPDH*) and uninfected cells. Results are presented as a heatmap (fold change, Log2), according to *IFNL4* genotype groups. **(D)** An increase in *DDIT3* expression at 24 hrs was significantly associated with *IFNL4* genotype, * p<0.05, linear trend. **(E)** Other UPR genes were not significantly associated with genotype, although they showed a similar trend as *DDIT3*.

**
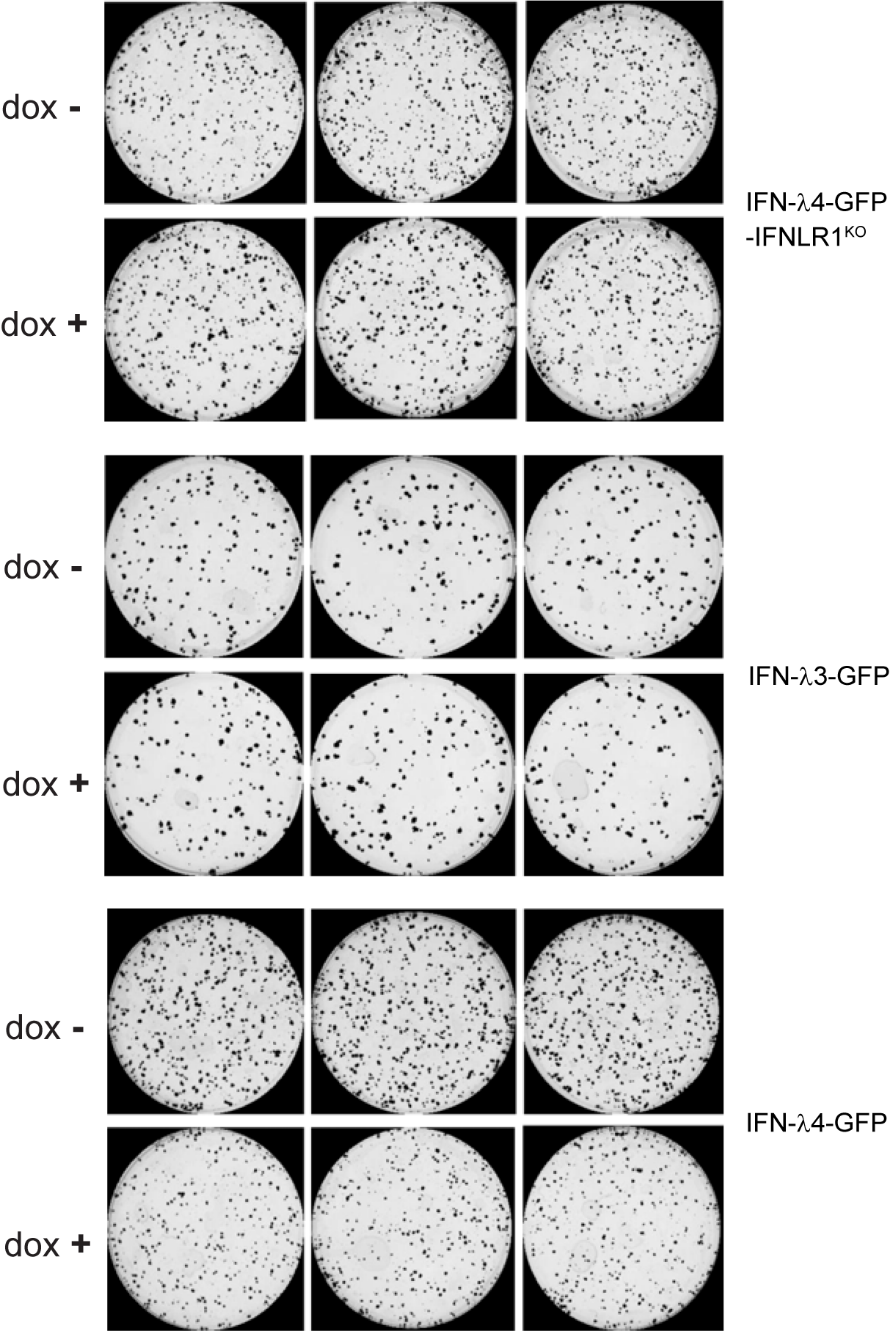
**

**Fig. S7. Inhibition of proliferation by IFN-λ4-GFP demonstrated by colony formation assay**

Images of IFN-λ3-GFP, IFN-λ4-GFP and IFN-λ4-GFP-IFNLR1^KO^ HepG2 cells grown in 6-well plates at a density of 5000 cells/well. The cells were dox-induced for 2 weeks, fixed, stained with crystal violet, and counted using ImageJ. The results are presented as plots in **Fig. 2F**.

**Supplementary Videos**

**Video S1**. Trafficking of IFN-λ4 in late endosomes in HepG2 cells.

HepG2 cells were transiently transfected with expression constructs IFN-λ4-Halo and GFP-Rab7a (late endosome marker). Cells were stained with a cell-permeant TMR Halo-tag ligand (red) and live-imaged starting 24 hrs post-transfection. Images were collected every minute for a total of 12 hrs. Snapshots of this video are presented in **Fig. 2B**. Video length: 12 sec

**Video S2**. Apoptosis of HepG2 cells expressing IFN-λ4.

HepG2 cells were transiently transfected with the IFN-λ4-Halo expression construct and live-imaged as described in **Video S1**. Video shows significant membrane blebbing immediately before cell rupture. Snapshots of this video are presented in **Fig. 2D**. Video length: 80 sec
